# Levels of circulating kidney injury markers and IL-10 identify non-critically ill patients with COVID-19 at risk of death

**DOI:** 10.1172/jci.insight.198244

**Published:** 2026-01-23

**Authors:** Olivia Lenoir, Florence Morin, Anouk Walter-Petrich, Léa Resmini, Mohamad Zaidan, Nassim Mahtal, Sophie Ferlicot, Victor G. Puelles, Nicola Wanner, Julien Dang, Thibaut d’Izarny-Gargas, Jana Biermann, Benjamin Izar, Stéphanie Baron, Benjamin Terrier, Ziad A. Massy, Marie Essig, Aymeric Couturier, Olivia May, Xavier Belenfant, David Buob, Isabelle Brocheriou, Hassan Izzedine, Yannis Lombardi, Hélène François, Anissa Moktefi, Vincent Audard, Aurélie Sannier, Eric Daugas, Matthieu Jamme, Guylaine Henry, Isabelle Le Monnier de Gouville, Catherine Marie, Laurence Homyrda, Céline Verstuyft, Sarah Tubiana, Ouifiya Kafif, Valentine Piquard, Maxime Dougados, Tobias B. Huber, Marine Livrozet, Jean-Sébastien Hulot, Cedric Laouénan, Jade Ghosn, France Mentré, Alexandre Karras, Yazdan Yazdanpanah, Raphaël Porcher, Philippe Ravaud, Sophie Caillat-Zucman, Xavier Mariette, Olivier Hermine, Matthieu Resche-Rigon, Pierre-Louis Tharaux, CORIMUNO-19 collaborative group

**Affiliations:** 1 Université Paris Cité, INSERM, Paris Cardiovascular research Center (PARCC), Paris, France.; 2 Laboratoire d’Immunologie et d’Histocompatibilité, and; 3 Biostatistics and Medical Information Department, Saint-Louis Hospital, Assistance Publique Hôpitaux de Paris, Paris, France.; 4 Department of Nephrology and Transplantation, Bicêtre Hospital, Assistance Publique Hôpitaux de Paris, Université Paris-Saclay, Le Kremlin-Bicêtre, France.; 5 Center for Rare Diseases, “Idiopathic Nephrotic Syndrome,” Bicêtre Hospital, Le Kremlin-Bicêtre, France.; 6 Department of Pathology, Bicêtre Hospital, Assistance Publique Hôpitaux de Paris, Université Paris-Saclay, Le Kremlin-Bicêtre, France.; 7 III. Department of Medicine, and; 8 Hamburg Center for Kidney Health (HCKH), University Medical Center Hamburg-Eppendorf, Hamburg, Germany.; 9 Program for Mathematical Genomics, Columbia University, New York, New York, USA.; 10 Department of Medicine, Division of Hematology/Oncology, and Herbert Irving Comprehensive Cancer Center, and; 11 Columbia Center for Translational Immunology, Columbia University Irving Medical Center, New York, New York, USA.; 12 Université Paris Cité, Sorbonne Université, INSERM, CNRS, Centre de Recherche des Cordeliers, Paris, France.; 13 Physiology Department, Assistance Publique Hôpitaux de Paris, Georges Pompidou European Hospital, Paris, France.; 14 Department of Internal Medicine, National Reference Center for Rare Systemic Autoimmune Diseases, Assistance Publique Hôpitaux de Paris, Cochin Hospital, Paris.; 15 Department of Nephrology, Ambroise Paré Hospital, Assistance Publique Hôpitaux de Paris, Boulogne Billancourt, France.; 16 Department of Nephrology, CHI André Grégoire, Montreuil, France.; 17 Department of Pathology, Assistance Publique Hôpitaux de Paris, Hôpital Tenon, Sorbonne Université, Paris, France.; 18 Sorbonne Université, Assistance Publique Hôpitaux de Paris, Pitié-Salpêtriere Hospital, Department of Cytology and Pathology, Paris, France.; 19 Department of Nephrology, Hôpital privé des Peupliers, Ramsay Générale de Santé, Paris, France.; 20 Sorbonne Université, INSERM, Assistance Publique Hôpitaux de Paris, Tenon Hospital, Nephrology Department, SINRA Unit, Paris, France.; 21 Department of Pathology, Henri Mondor Hospital, Assistance Publique Hôpitaux de Paris, Paris-Est-Créteil University, Paris, France.; 22 Department of Nephrology and Transplantation, Assistance Publique Hôpitaux de Paris, Henri Mondor Hospital, Reference Center for Rare Diseases, “Idiopathic Nephrotic Syndrome,” University Hospital Federation, “Innovative Therapy for Immune Disorders,” Créteil, France.; 23 Université Paris Est Créteil, Institut National de la Santé et de la Recherche Médicale (INSERM) U955, Institut Mondor de Recherche Biomédicale (IMRB), Créteil, France.; 24 Université Paris Cité, Department of Pathology, and; 25 Université Paris Cité, Department of Nephrology, Bichat Hospital, Assistance Publique Hôpitaux de Paris, Paris, France.; 26 Department of Intensive Care Medicine, Centre Hospitalier Intercommunal de Poissy, Poissy, France.; 27 Department of Molecular Genetics, Pharmacogenomics and Hormonology, Bicêtre Hospital, Paris Saclay Biological Resource Center, Le Kremlin-Bicêtre, France.; 28 Biological Resource Center, Bichat - Claude Bernard Hospital, Assistance Publique Hôpitaux de Paris, Paris, France.; 29 Université Paris Cité, INSERM, IAME UMR1137, Paris, France.; 30 Université Paris Cité, Department of Rheumatology, Cochin Hospital, Assistance Publique Hôpitaux de Paris, Paris, France.; 31 INSERM, Clinical Epidemiology and Biostatistics, PRES Sorbonne Paris-Cité, Paris, France.; 32 Clinical Investigation Center CIC1418 and DMU CARTE, Georges Pompidou European Hospital, Assistance Publique Hôpitaux de Paris, INSERM, Paris, France.; 33 Department of Epidemiology, Biostatistics and Clinical Research, Bichat - Claude Bernard Hospital, Assistance Publique Hôpitaux de Paris, INSERM, Centre d’Investigation Clinique Epidemiologie 1425, Paris, France.; 34 Department of Infectious and Tropical Diseases, Bichat - Claude Bernard Hospital, Assistance Publique Hôpitaux de Paris, Paris, France.; 35 Department of Nephrology, Georges Pompidou European Hospital, Assistance Publique Hôpitaux de Paris, Université Paris Cité, Paris, France.; 36 ANRS Maladies Infectieuses Émergentes, Paris, France.; 37 REACTing, Institut Thématique Immunologie, Inflammation, Infectiologie et Microbiologie, INSERM, Paris, France.; 38 Centre for Research in Epidemiology and Statistics (CRESS), INSERM, Inrae, Université Paris Cité, Paris, France.; 39 Center for Clinical Epidemiology, Hôtel-Dieu Hospital, Paris, France.; 40 INSERM UMR976, Human Immunology, Pathophysiology and Immunotherapy, Université Paris Cité, INSERM, Paris, France.; 41 Department of Rheumatology, INSERM UMR 1184: Center for Immunology of Viral Infections and Autoimmune Diseases, Bicêtre Hospital, Assistance Publique Hôpitaux de Paris, Université Paris-Saclay, Le Kremlin-Bicêtre, France.; 42 Department of Hematology, Necker Hospital, Assistance Publique Hôpitaux de Paris, Université de Paris, Paris, France.; 43 Laboratory of Pathophysiology and Treatment of Hematological Malignancies, Institut Imagine, INSERM U1153, Université de Paris, Paris, France.; 44 The CORIMUNO-19 collaborative group is detailed in Supplemental Acknowledgments.

**Keywords:** Infectious disease, Nephrology, Pulmonology, COVID-19, Clinical trials, Outcomes research

## Abstract

**BACKGROUND:**

After identifying 2 immunomarkers of acute injury, KIM-1 and LCN2, in all kidney biopsies from 31 patients with COVID-19 pneumonia and de novo kidney dysfunction, we investigated whether circulating markers of kidney epithelial injury are common in patients with laboratory-confirmed COVID-19 who require oxygen support but do not have critical illness.

**METHODS:**

We studied 196 patients admitted to 15 hospitals with moderate to severe pneumonia who were enrolled in 2 independent randomized clinical trials. We measured 41 immune mediators and markers of kidney and endothelial injury in peripheral blood in these patients within 24 hours of randomization.

**RESULTS:**

We constructed a generalized linear CORIMUNO model combining serum levels of KIM-1, LCN2, IL-10, and age at hospital admission that showed high discrimination for mortality (derivation cohort: AUC = 0.82, 95% CI: 0.73–0.92; validation cohort: AUC = 0.83, 95% CI: 0.74–0.92). An early rise in circulating kidney injury markers, in the absence of acute kidney injury criteria, was markedly associated with the risk of developing a severe form of COVID-19 and death within 3 months.

**CONCLUSION:**

The CORIMUNO score may be a helpful tool for risk stratification, and for the first time to our knowledge, it identifies the overlooked impact of subclinical kidney injury on pneumonia outcomes.

**TRIAL REGISTRATION:**

ClinicalTrials.gov NCT04324047, NCT04324073, and NCT04331808.

**FUNDING:**

This research was funded by the French Ministry of Health, Programme Hospitalier de Recherche Clinique (PHRC COVID-19–20–0151, PHRC COVID-19–20–0029), Fondation de l’Assistance Publique Hôpitaux de Paris (Alliance Tous Unis Contre le Virus), Assistance Publique Hôpitaux de Paris, and grants from the Fondation pour la Recherche Médicale (FRM) (REA202010012514) and Agence Nationale de Recherches sur le Sida and emerging infectious diseases (ANRS) (ANRS0147) from the VINTED sponsorship.

## Abstract 2

A strong association between kidney-derived proteins and IL-10 levels and mortality supports the notion that the kidney is an overlooked sentinel organ in SARS-CoV-2 pneumonia, even in the absence of overt acute kidney injury.

## Introduction

In January 2020, the WHO declared COVID-19 a public health emergency. During the early epidemic waves before vaccines, kidney involvement was frequent, and over 20% of hospitalized patients with COVID-19 developed acute kidney injury (AKI). Most studies focused on critically ill patients with severe AKI (1–4). Studies have shown similarities between sepsis-associated AKI and COVID-19–associated AKI (5, 6), and critically ill patients with COVID-19 are at increased risk of severe AKI (7), defined as Kidney Disease Improving Global Outcomes (KDIGO) stages 2 or 3 (8). Recent literature shows AKI incidence in hospitalized patients with COVID-19 ranges from 8% to 22% (9). Prognosis and kidney injury marker studies have mostly focused on critically ill patients with cytokine-storm phenotypes and coagulation disorders, and few multicenter studies have been conducted on moderate to severe pneumonia. Therefore, we investigated whether circulating markers of kidney epithelial acute injury are present in patients with laboratory-confirmed COVID-19 on oxygen support but without critical pneumonia or AKI.

Meta-analyses of clinical trials for COVID-19 have had the challenge of clearly defining populations enrolled in clinical trials, as they differ markedly in terms of mortality, despite efforts to standardize the assessment of clinical progression scores by the WHO and empirical comparisons of routine inflammatory markers and comorbidities. Thus, given the uncertainty surrounding the stratification of patients with COVID-19, considerable interest exists in risk stratification scores to support frontline clinical decision-making. However, current tools have high bias risk and limited sample sizes, which lead to uncertainty and limited formal validation. To overcome these gaps, we performed multiparameter molecular analyses in well-defined cohorts and validated findings independently. Such molecular analyses encompassed markers of kidney tubular injury, systemic inflammation, and endothelial alterations.

However, no study has integrated data from readily accessible samples, such as serum or plasma, obtained from well-characterized COVID-19 patients and control individuals, while systematically excluding key confounding factors, including age, sex, and comorbidities, from the outset. Although AKI correlates strongly with poor prognosis in critical intensive care unit (ICU) cases and non–COVID-19 sepsis (1, 3, 7, 10–12), we explored kidney alterations in adult patients hospitalized with moderate or severe COVID-19 pneumonia, even in the absence of AKI criteria.

First, we evaluated the prevalence of markers of kidney epithelial injury in routine biopsies of patients with moderate or severe COVID-19 pneumonia with AKI or proteinuria. Next, given the pleiotropic nature of SARS-CoV-2 sepsis and because most published work has focused on a specific biological system, we thought that a combination of multiple biological molecules may capture pathomechanisms or organ failure. Therefore, we evaluated whether an early measurement of a panel of serum markers for inflammation, endothelial injury, and kidney tubular injury was associated with the risk of critical COVID-19 pneumonia and death across 15 hospitals in France. We tested the hypothesis that combining markers and mediators of a SARS-CoV-2–induced hyperinflammatory state with endothelial and renal injury could help identify patients with COVID-19 at increased risk of dying within 90 days of hospitalization.

First, we observed a constant upregulation of kidney injury molecule-1 (KIM-1) and lipocalin-2 (LCN2) proteins in kidneys of patients with overt nephropathy, that is, with proteinuria or impaired glomerular filtration rate (GFR) during SARS-CoV-2 infection. When extending our investigation to patients with moderate to severe COVID-19 pneumonia not requiring an ICU stay, as defined by the 10-point WHO clinical progression scale (13), we were surprised to observe that among 41 immune mediators and kidney and endothelial injury markers in the blood, elevated IL-10, KIM-1, and LCN2 levels were significantly associated with 90-day mortality, even in the absence of AKI criteria, including in patients with normal GFR. A prognostic index was derived using penalized logistic regression (least absolute shrinkage and selection operator [LASSO]) and validated on an independent cohort. The resulting generalized CORIMUNO model combined age with IL-10, KIM-1, and LCN2 levels, revealing significant mortality discrimination. These markers outperformed inflammatory cytokines to identify patients at risk of death, highlighting the critical impact of subclinical kidney injury on outcomes in viral sepsis.

## Results

### Immunostaining of AKI markers in patients with COVID-19 with moderate to severe pneumonia.

**Figure 1:**
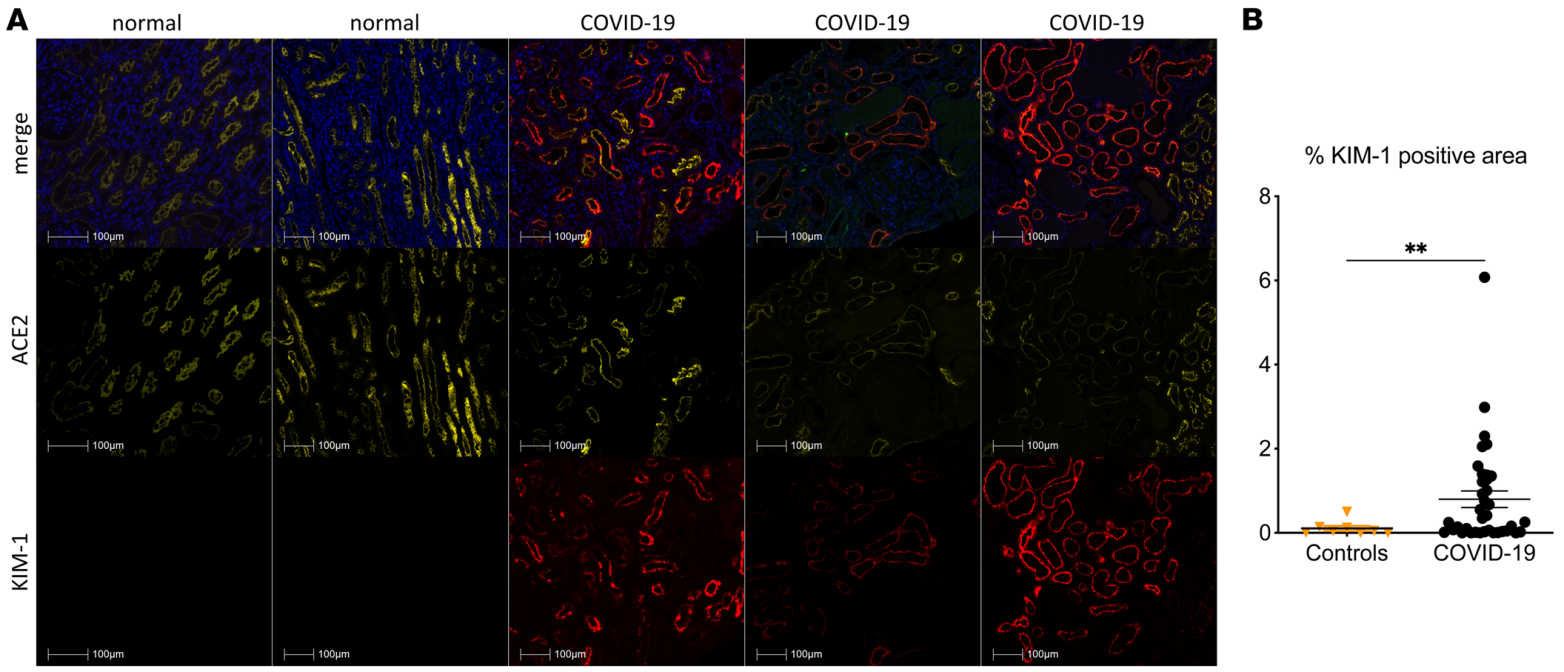
KIM-1 expression in kidneys of patients with COVID-19. (**A**) Representative photomicrographs of KIM-1 (red) and ACE2 (yellow) immunostaining of 3 cases of COVID-19–related acute kidney injury and 2 optically normal kidney sections. (**B**) Comparison of KIM-1–associated immunoreactive signal in whole slide images of kidney biopsies of 31 patients with COVID-19 and 8 controls. **P < 0.01 versus controls. Scale bars: 100 μm.

**Figure 2:**
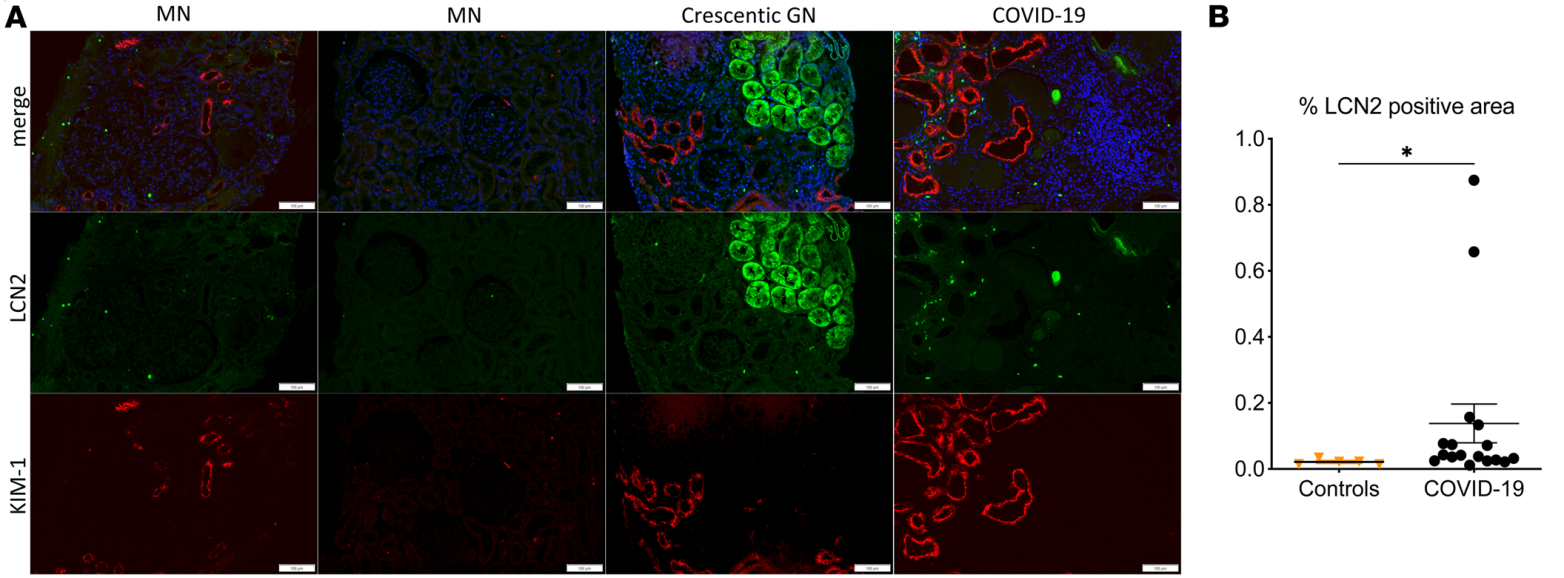
LCN2 expression in kidneys of patients with COVID-19. (**A**) Representative photomicrographs of LCN2 (green) and KIM-1 (red) immunostaining of 1 case of COVID-19–related acute kidney injury and 3 nephropathies as controls. Membranous nephropathy (MN) and crescentic glomerulonephritis (crescentic GN) anti-neutrophil cytoplasm antibody–associated (ANCA-associated) were used as positive controls for LCN2 staining. (**B**) Comparison of LCN2-associated immunoreactive signal in whole slide images of kidney biopsies of 17 patients with COVID-19 and 5 controls. *P < 0.05 versus controls. Scale bars: 100 μm.

We first examined whether non-critically ill patients with COVID-19 with clinically overt kidney injury (KDIGO stage 1 and 2 acute AKI or with no AKI but overt proteinuria) would display characteristics of kidney tubular injury. We performed KIM-1 immunostaining in 31 kidney biopsies from patients with COVID-19 who showed acute alteration of kidney function or proteinuria that unraveled marked overexpression of KIM-1 protein in kidney tubular cells (Figure 1). Of the 31 kidney biopsies, 29 (94%) displayed detectable tubular expression of KIM-1 that was not observed in 2 normal kidneys diagnosed with sarcoidosis and 6 kidneys from patients with non–COVID-19 membranous nephropathy (2 kidneys) or minimal change disease (4 kidneys). Surprisingly, ACE2 expression, a receptor for SARS-CoV-2, was generally milder in tissues of patients with COVID-19 than in controls, suggesting downregulation of ACE2 concomitant with overexpression of KIM-1. KIM-1–associated signal extended beyond ACE2-positive tubular segments (Figure 1). We also found increased expression of LCN2, another marker of tubular injury (14), in 17 kidney biopsies of patients with COVID-19 (Figure 2). LCN2 was not found in normal kidneys (not shown) and in 5 cases not associated with COVID-19 (proteinuric membranous nephropathy and minimal change disease) (Figure 2).

### Determination of tubular injury and identification of cellular sources of AKI markers in COVID-19 in postmortem samples.

**Figure 3:**
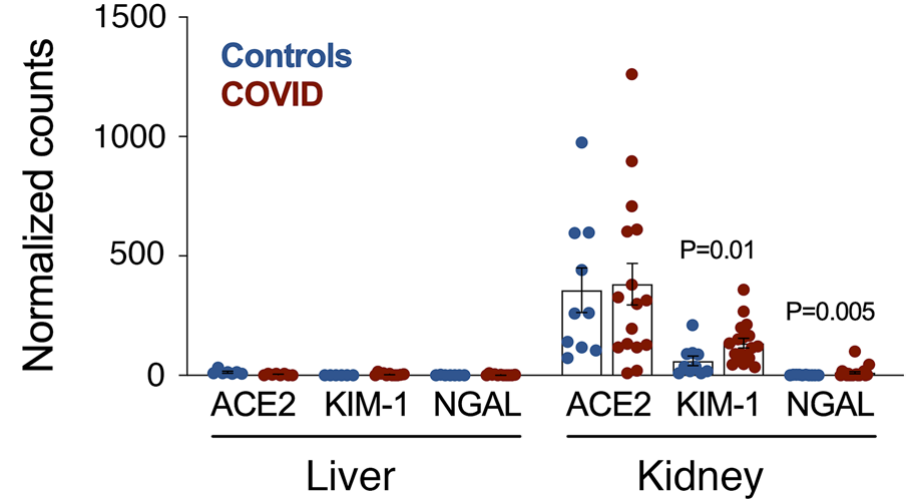
Comparison of KIM-1, LCN2/NGAL, and ACE2 mRNA abundance in autopsy kidneys and liver from COVID-19 critical cases and controls. The data are expressed as DESeq2-normalized counts.

We also ran an RNA-seq analysis in 20 kidneys and 11 livers from the autopsy of deceased patients with COVID-19 and in 7 control livers and 10 control kidneys (non–COVID-19 autopsy). We found a significantly increased abundance of *HAVCR1* mRNA (encoding the KIM-1 protein) in the kidneys of patients with COVID-19 but not in the liver (Figure 3). We could also confirm an increase in *LCN2* mRNA abundance in the kidneys of these patients with COVID-19. By contrast, the SARS-CoV-2 receptor *ACE-2* was not differentially expressed in these tissues of deceased patients with COVID-19, as well as *IL-6*, IL-6 cytokine family signal transducer (*IL-6ST*), IL-1 receptor antagonist (*IL-1RN*), and *IL-10* mRNA (not shown).

### Determination of the cellular source of kidney injury–associated markers in tissues from deceased patients with COVID-19.

To validate our findings and refine the cellular origin of KIM-1 and LCN2 in COVID-19 conditions, we reviewed publicly available single-cell RNA-seq (scRNA-seq) datasets from the kidneys of patients with COVID-19 (15–17). We evaluated the relative abundance of the following markers of kidney injury: *HAVCR1*; *LCN2*; plasminogen activator urokinase receptor (*PLAUR*)*,* also known as urokinase plasminogen activator surface receptor (uPAR); osteopontin/secreted phosphoprotein 1 (*SPP1*); and trefoil factor 3 (*TFF3*). In addition, we conducted a postmortem evaluation of *IL-6* and *IL-10* mRNA in kidney cell types from 16 patients deceased from COVID-19 critical pneumonia (16). As observed at the protein level by immunofluorescence, we found that among cell populations in the kidney, *HAVCR1* mRNA was expressed at much higher levels in kidney tubular epithelial cells (TECs) than in immune cells and other resident cells, indicating that kidney TECs are the primary cell source of KIM-1 production in patients with COVID-19 (Supplemental Figure 1; supplemental material available online with this article; https://doi.org/10.1172/jci.insight.198244DS1), from parietal epithelial cells to the thick ascending limb. Among other markers of AKI, *LCN2* was also found to be expressed by kidney epithelial cells, *PLAUR* by myeloid cell subsets, *SPP1* by parietal epithelial cells, and all the tubular segments from the proximal convoluted tubule to the collecting duct with some macrophages, and *TFF3* was expressed by renal lymphatic endothelial cells. *IL-6* was expressed by some endothelial cells, pericytes, and fibroblasts; some macrophages and monocytes expressed *IL-10*.

In the lungs of patients with critical COVID-19 (Supplemental Figure 2), a low detectable level of *HAVCR1* mRNA was found in some T cells and antigen-presenting cells but not in epithelial cells, as opposed to the kidney. *LCN2* expression was found in TECs and myeloid cells, whereas *PLAUR* mRNA was markedly abundant in myeloid and mast cells, and to a lesser extent in antigen presenting–like cells. *SPP1* mRNA was not significantly expressed in epithelial cells but was expressed in myeloid cells and some antigen-presenting cells. *TFF3* was detected in neuronal and tuft-like cells. As in the kidneys, *IL-6* was detected in some endothelial cells, pericytes, and fibroblasts. *IL-10* was expressed by some myeloid and T cells in the lungs.

The findings were confirmed in another public dataset, which showed high expression of *HAVCR1* in parietal epithelial cells, proximal convoluted tubules, the thick ascending limb, the thin ascending limb, and some expression in connecting tubules and distal convoluted tubules. Limited HAVCR1 mRNA expression was observed in certain NK cells, T regulatory lymphocytes, and macrophages in the lungs. Additionally, *LCN2* mRNA was expressed in the kidney by parietal epithelial cells, the thick ascending limb, the thin ascending limb, and in some connecting tubules (https://singlecell.broadinstitute.org/single_cell/study/SCP1214/covid-19-kidney-autopsy-samples?genes=HAVCR1&cluster=UMAP&spatialGroups=--&annotation=predicted_celltype--group--study&subsample=all&tab=distribution&distributionPlot=violin&distributionPoints=all#study-visualize; and https://singlecell.broadinstitute.org/single_cell/study/SCP1214/covid-19-kidney-autopsy-samples?genes=LCN2&cluster=UMAP&spatialGroups=--&annotation=predicted_celltype--group--study&subsample=all&tab=distribution&distributionPlot=violin&distributionPoints=all#study-visualize).

Overall, our findings demonstrated that kidney epithelial cells in patients with COVID-19 pneumonia expressed a large amount of KIM-1/HAVCR1 and SPP1 mRNA and some LCN2 mRNA with lower abundance.

### Association of cytokines and endothelial and renal injury markers with death in 2 independent cohorts of patients.

We next investigated whether circulating markers of such acute kidney epithelial cell injury are frequent in patients with laboratory-confirmed COVID-19 requiring oxygen support. We studied 196 patients admitted to 15 hospitals in France with either mild (133 patients) or severe (63 patients) disease, enrolled in 2 independent randomized clinical trials (18–20). The patients’ characteristics are summarized in Tables 1 and 2. Patients were stratified into 3 clinical groups based on their peak illness severity at enrollment according to the WHO COVID-19 clinical progression scale (CPS; ref 13): (a) patients requiring oxygen by face mask or nasal prongs (CPS severity 5, *n* = 133); (b) patients requiring high-flow nasal cannula oxygen or noninvasive ventilation (severity 6, *n* = 15); (c) patients requiring invasive mechanical ventilation (severity > 6, *n* = 48) (Table 3).

**Figure 4:**
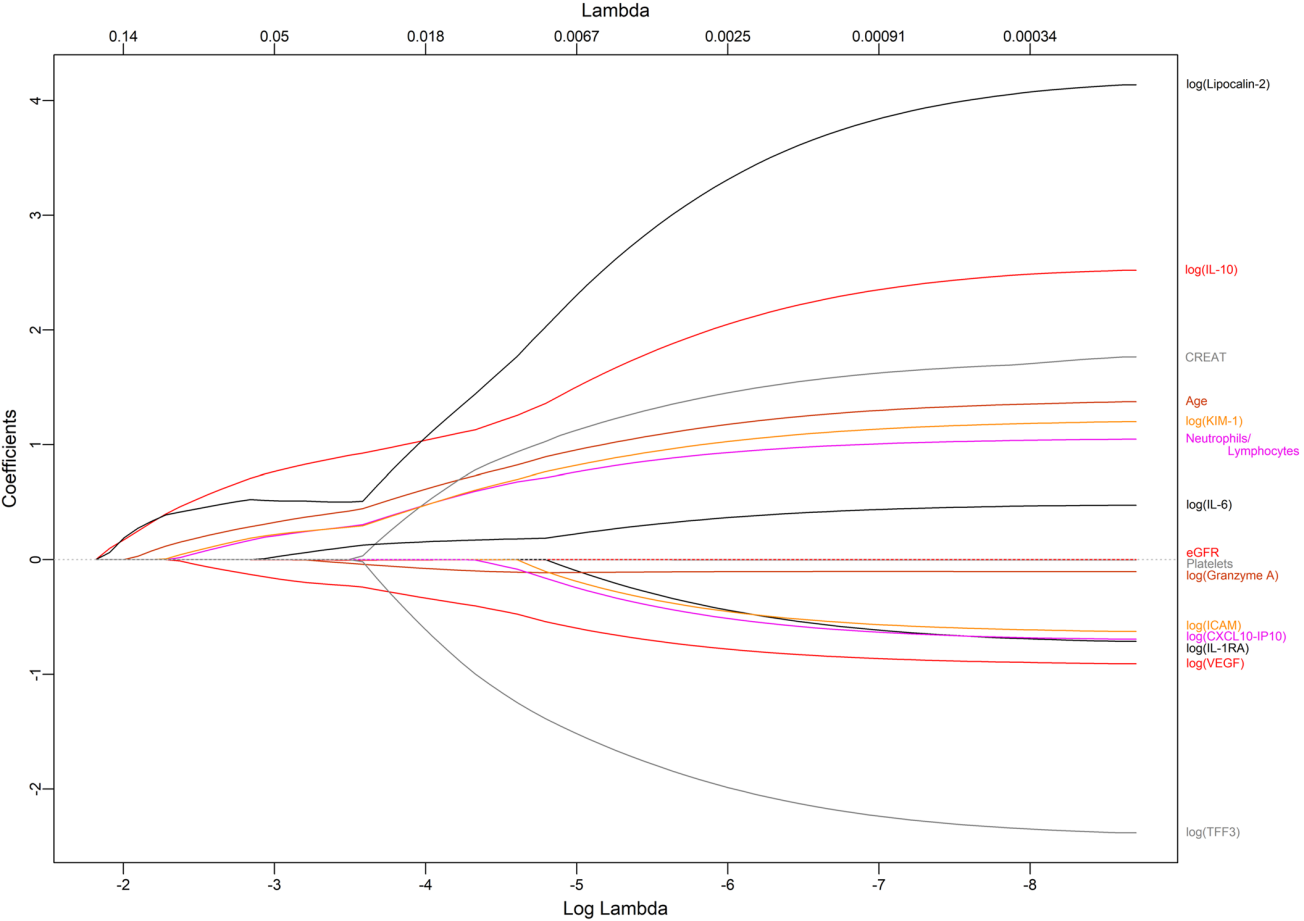
Feature selection using the LASSO binary logistic regression model and multivariable analysis in the training cohort. A logistic regression was performed using the selected variables (see Table 5). Lambda = 0.10.

In these patients with COVID-19 pneumonia, we measured 41 immune mediators, cytokines, and markers of kidney and endothelial injury in peripheral blood (Supplemental Table 1). Ninety-one patients from the CORIMUNO-SARI trials (training cohort) had measurements taken on day 1 of randomization. Age at randomization and 14 biological variables (including 11 proteins) were independently associated with the risk of death within 90 days (*P* < 0.05, 17 with *P* < 0.15) (Table 4). Initially, to include as many patients as possible (*n* = 75, 19 deaths), only the following variables were retained: age, platelet count, neutrophils/lymphocytes, creatinine, estimated GFR (eGFR), log(KIM-1), log(IL-1RA), log(IL-6), log(IL-10), log(ICAM-1), log(granzyme A), log(VEGF), log(CXCL10.IP10), log(lipocalin-2), and log(TFF3) (Figure 4).

**Figure 5:**
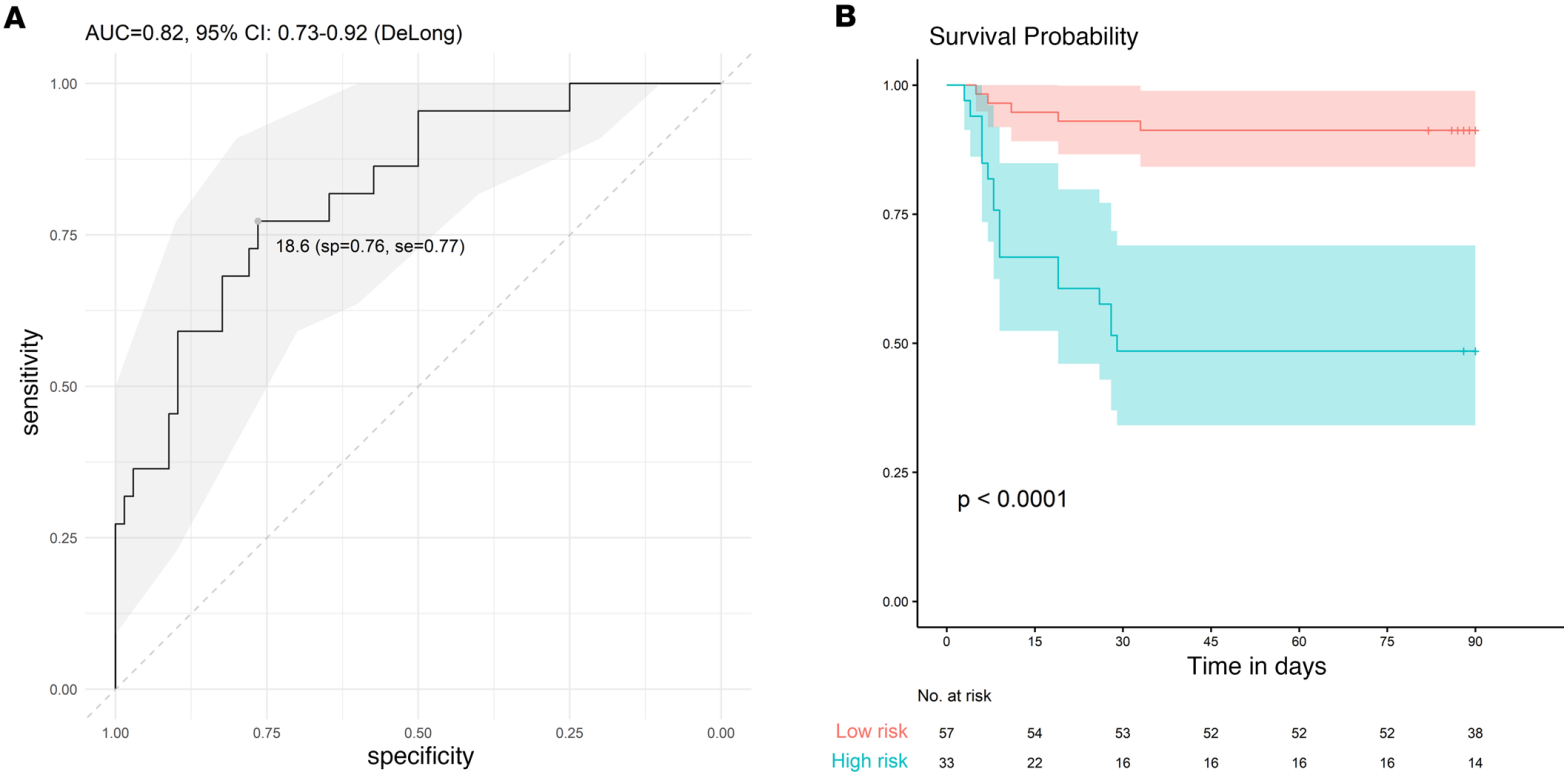
Performance of the scoring system to predict mortality of patients with COVID-19. (**A**) ROC curve and AUC to assess the prediction accuracy in the training cohort. Youden index: 18.6. A threshold of 16.9 corresponded to a 98% negative predictive value, and a threshold of 20.9 corresponded to an 87% positive predictive value. (**B**) Kaplan-Meier survival curves with 95% CIs for high- and low-risk groups in the training cohort. sp, specificity; se, sensitivity.

A generalized linear model, performed with 90 patients (1 missing value for 1 patient, 22 deaths) from the CORIMUNO-SARI trials, retained 4 variables associated with odds ratios for the risk of coronavirus-related deaths ranging from 1.08 to 4.41 (Table 5). The following model was computed: y = β1 × AGE + β2 × log(KIM-1) + β3 × log(IL-10) + β4 × log(LCN2). The ROC with associated 95% CI was calculated in the training cohort with AUC = 0.82, 95% CI: 0.73–0.92 (Figure 5A). We then determined the Youden index for the ROC (18.6, specificity [sp] = 0.76, sensitivity [se] = 0.77), which enabled discrimination between 2 groups with very significantly different outcomes: a low-risk group (score < 18.6) and a high-risk group (score ≥ 18.6) (Figure 5B). The CORIMUNO score discriminated between 2 groups of patients with a low or high risk of death, with an odds ratio of 11.05 (3.52–34.68); *P* < 0.0001 compared with the low-risk group.

### Validation of the CORIMUNO score in an independent cohort.

**Figure 6:**
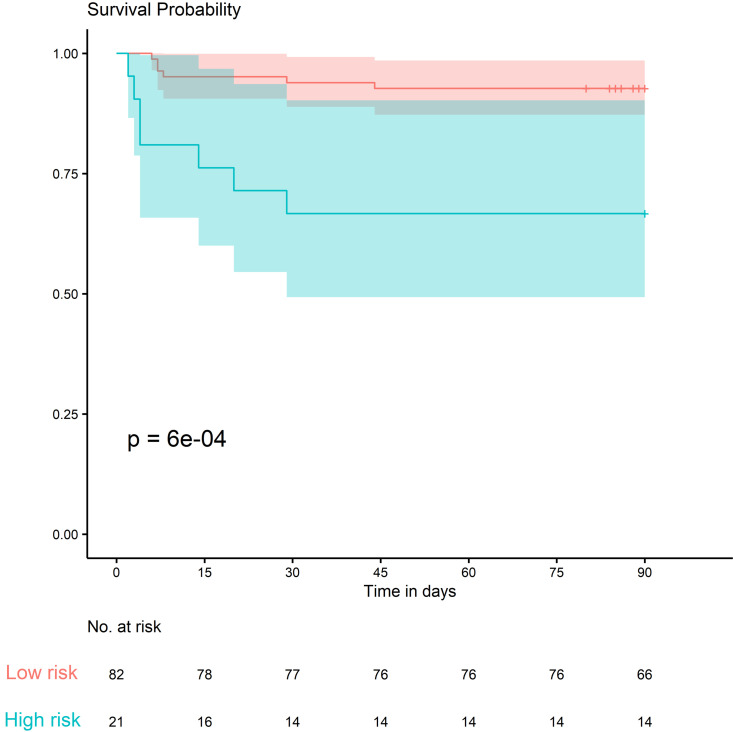
Kaplan-Meier survival curves with 95% CIs for high- and low-risk groups in the validation cohort.

Next, we validated the score in a sample of 105 individuals enrolled in the independent CORIMUNO-TOCI trial. Using univariate analysis, we found that age and 10 biological parameters were associated with death at a significance threshold of *P* < 0.05 (Table 6). Interestingly, despite having similar inclusion criteria and mean age at inclusion, and completing the trials within a relatively narrow timeframe, the 2 patient populations shared certain parameters that were independently associated with the risk of death, although not all. These common independent risk factors for death within 90 days were age, KIM-1, IL-6, IL-10, and, to a lesser extent, granzyme A, IL-1RA, lipocalin2, and TFF3 circulating concentrations, each being significantly more elevated in the group with the lethal outcome (Tables 4 and 6). We computed the model above using this validation cohort and obtained an AUC of 0.83 (95% CI: 0.74–0.92). We identified 2 groups with very different outcomes by day 90 (Figure 6), indicating the robustness of the model. The high-risk group had an odds ratio of 6.33 (1.85*–*21.68) compared with the low-risk group (*P* = 0.003). When computed across the entire patient population, including both cohorts, the positive predictive value was 44.4% and the negative predictive value was 92.1%. A threshold of 16.9 corresponded to a 98% negative predictive value, and a threshold of 20.9 corresponded to an 87% positive predictive value.

### Comparison of score performance in patients receiving usual care and patients receiving anti–IL-6R Ab.

**Figure 7:**
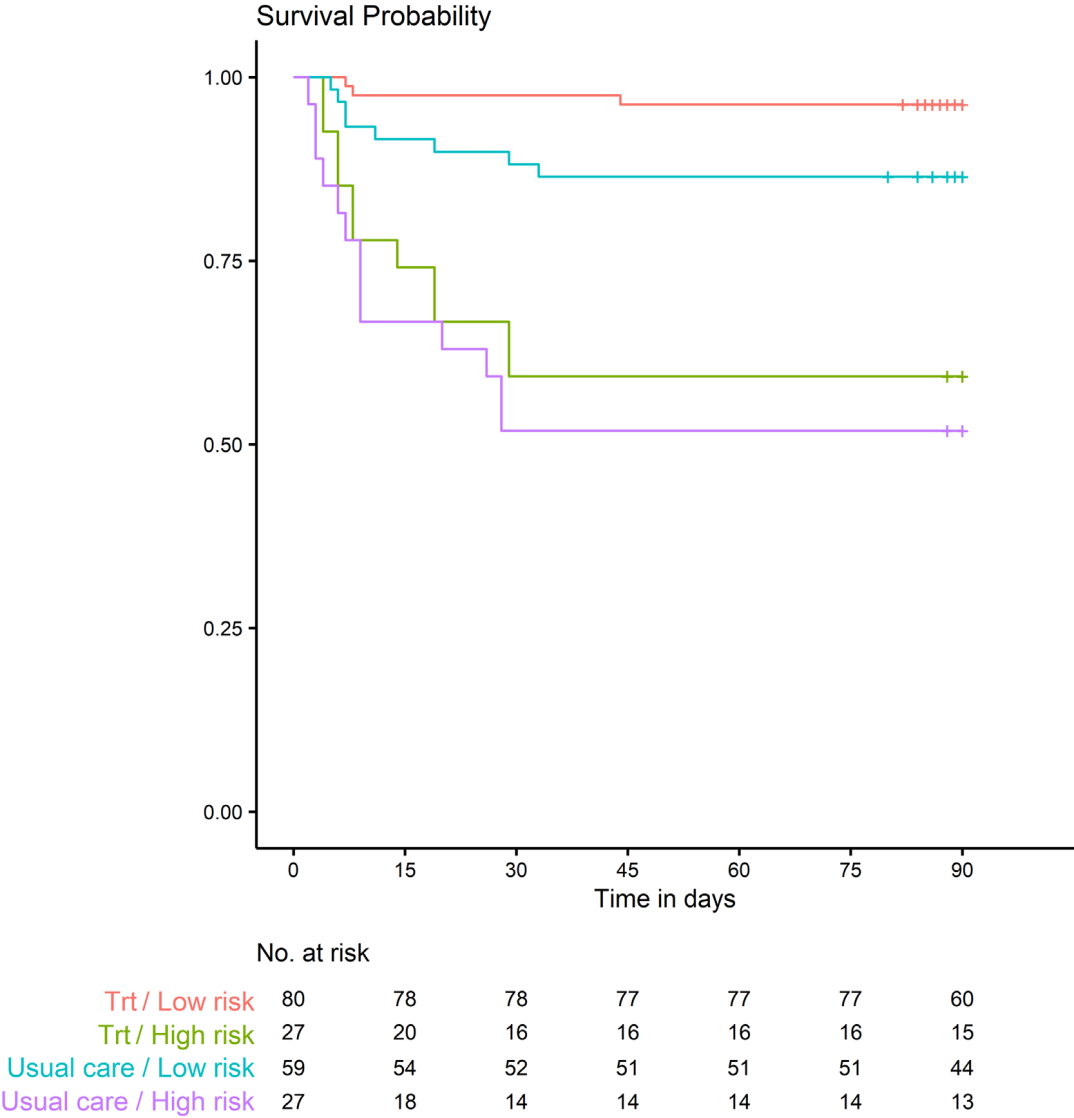
Kaplan-Meier survival curves of patients with COVID-19 stratified into low- and high-risk groups in the anti–IL-6R Ab treatment arms (Trt) and the usual care arms.

Next, because serum IL-6 concentration at the time of early hospitalization was associated with both the risk of worsening of WHO CPS at day 14 (Supplemental Table 2) and the risk of death within 90 days (Tables 4 and 6, and Supplemental Table 3), we evaluated whether the model could still discriminate patients’ outcomes irrespective of anti–IL-6R treatment. This remained the case when comparing high-risk and low-risk mortality groups in patients treated with sarilumab or tocilizumab versus those in the usual care (UC) arms (Figure 7). Among patients treated with sarilumab or tocilizumab, the difference in survival between the high- and low-risk groups was highly significant (*P* < 0.0001).

In the UC group, the difference in survival between the high-risk and low-risk groups was also highly significant (*P* = 0.0002). Thus, the CORIMUNO score’s predictive capacity was effective irrespective of the treatment. In the low-risk group, but not in the high-risk group, the survival of patients in the treatment arm and patients in the UC arm was significantly different (*P* = 0.033 and *P* = 0.53, respectively).

### Evaluation of the influence of specific anti–IL-6R antibodies on the predictive capacity of the CORIMUNO score.

Given the robustness of the predictive score across both the anti–IL-6R Ab treatment and UC arms, we split the samples within each cohort to assess the score’s performance in patients treated with sarilumab versus tocilizumab and the UC arm. Again, the 18.6 Youden index discriminated between high- and low-risk patient groups within each randomized treatment condition (Supplemental Figure 3). Within each independent cohort, the difference in survival between the high-risk and low-risk groups was also significant in sarilumab-treated patients (*P* < 0.0001) and UC controls (*P* = 0.015). Likewise, the difference in survival between the high-risk and low-risk groups was also significant in patients treated with tocilizumab (*P* = 0.012) and their matched UC controls (*P* = 0.017). Mortality did not differ between the 2 randomization arms in the high-risk group (*P* = 0.45) and low-risk group (*P* = 0.23) (Supplemental Figure 4).

To decipher how the combination of markers of kidney injury contributed to risk prediction in addition to inflammation markers, we conducted a cluster dendrogram, correlation matrix, and principal component analysis to summarize and visualize the information in our datasets containing observations described by multiple intercorrelated quantitative variables (Supplemental Figures 5–7). The cluster dendrogram indicated the relationships between proinflammatory, antiinflammatory, endothelial, and kidney tubular injury markers. The principal component analysis showed that the main markers displayed low redundancy (Supplemental Figure 7). One quadrant brought together inflammation markers (IL-6, CRP, MCP-1, TNF-α, IL-1RA, IL-10, neutrophil count), whereas the characteristics of kidney-related molecules (KIM-1, LCN2, TFF3, uPAR, creatinine) were brought together in a distinct orthogonal quadrant. VEGF, PDGF-AA, PDGF-BB, and lymphocyte count were brought together in a separate quadrant, suggesting the contribution of vascular mediators, although sICAM-1, sVCAM-1, and sGp130 were in an intermediate situation. The various markers of tubular injury and GFR were imperfectly correlated, underscoring their added value (Supplemental Figures 6 and 8). Overall, this analysis indicates that inflammation, kidney, and vascular modules provide complementary information on outcome, regardless of anti–IL-6R therapy. The respective ROC curves and AUC values for each variable, along with their contribution to the combined score, are presented in Supplemental Figure 9.

Next, we wondered whether an enrichment bias in patients with AKI drove the high predictive value of the kidney markers. However, the vast majority of our patients were hospitalized in the ward; 62.64% and 72.38% of patients in the training and validation cohorts had moderate COVID-19 pneumonia, respectively, requiring only an O2 prong (WHO CPS 5). Nonetheless, we tested the model in 2 groups: one of patients with COVID-19 with eGFR ≥60 mL/min/1.73 m² and another with eGFR deterioration (<60 mL/min/1.73 m²) at the time of serum marker concentration assessment (Table 7). The CORIMUNO SCORE was effective in both groups but, surprisingly, achieved better discrimination between the high-risk and low-risk death groups in patients with normal eGFR (*P* < 0.0001) (Supplemental Figures 10 and 11).

Last, we compared the overall discrimination of our score with the 4C mortality score for COVID-19, produced by the ISARIC consortium (21), by calculating AUCs and testing the difference using DeLong’s test for correlated ROC curves. There was no evidence of a difference in discrimination between the CORIMUNO score and the ISARI-4C score (AUC = 0.82, 95% CI: 0.66–0.99 vs. AUC = 0.85, 95% CI: 0.64–1; DeLong *P* = 0.83). The main comparison between our score and the ISARI-4C score was performed in the independent validation cohort to avoid overoptimism. For completeness, we also repeated the comparison in the overall cohort below (DeLong *P* = 0.22) (Supplemental Figure 12).

## Discussion

In this study, we demonstrate that integrating circulating markers of kidney tubular injury yields a simple predictive score for clinical worsening and death in patients with moderate COVID-19 pneumonia, even in the absence of AKI criteria. This study indicates a significant prevalence of unnoticed kidney tubular injury, characterized by elevated blood concentrations of specific markers in cohorts where most patients (68%) were hospitalized in the ward (WHO progression score <6). Among 41 circulating markers measured early after hospitalization, 15 were independently associated with 90-day mortality. Dimensionality reduction with LASSO regression enabled the establishment of a predictive mortality risk score, which was validated in an independent multicenter cohort.

We aimed to develop and validate a straightforward, biologically relevant risk score combining a limited set of parameters, which could be implemented at hospital presentation. Prior studies have reported the molecular and cellular modifications associated with COVID-19 severity (22–33), but they used hard-to-generalize and labor-intensive methods. Furthermore, the few that used simpler measurements focused on critically ill patients and were limited to epidemiology and/or standard bioclinical parameters such as CRP, neutrophil to lymphocyte ratio, LDH, D-dimers, and APACHE II or Sequential Organ Failure Assessment (SOFA) scores, achieving moderate performance (21, 34–38). The SOFA score is of limited utility because patients with COVID-19 pneumonia generally have severe single-organ dysfunction and exhibit less variation in SOFA scores. The more practical and well-validated in-hospital 4C mortality score outperformed several other scores (21, 38). Initially, the 4C 8-variable score showed high discrimination for mortality (derivation: AUROC 0.79, 95% CI: 0.78–0.79; validation: 0.77, 95% CI: 0.76–0.77) (21). The CORIMUNO score, which combines 3 biological variables and age at a single time point, achieved a comparable AUROC (0.82 and 0.83 in the training and validation cohorts, respectively; AUC = 0.83 [95% CI: 0.76–0.89] in the pooled sample). Interestingly, there was no evidence of a difference in discrimination between the CORIMUNO score and the ISARI-4C score in our cohort, suggesting that the simple combination of 4 variables in our score captures the various complex comorbidities and clinical status integrated by ISARI-4C.

Using an independent external dataset improved the generalizability of our results. Nonetheless, a limitation of this study is the limited sample size. We acknowledge that the external validation dataset was slightly above 100 events, the recommended minimum (39). Substantial effective sample sizes were required for external validation studies of predictive logistic regression models (40).

We confirmed that severe COVID-19 correlates with elevated coagulation and inflammatory molecules — CXCL10, MCP-1, IL-8, IL-10, IL-6 — mirroring monocentric findings (41). Another monocentric study confirmed that inhibitory cytokines IL-1RA and IL-10 are significantly elevated in severe cases at the early stage of infection (42). Systemic inflammation with high levels of acute-phase proteins (CRP, SAA, calprotectin) (29) and inflammatory cytokines, particularly IL-6 and IL-1β (25, 43, 44), is a hallmark of disease severity along with sustained high viral load (45–47). Overall, our multicentric prospective study confirmed findings from several monocentric studies that both mild and severe forms of the disease result in changes in circulating leukocyte subsets and cytokine secretion, particularly IL-6, IL-1β, IL-10, TNF-α, GM-CSF, IP-10 (IFN-induced protein 10), IL-17, MCP-3, and IL-1RA (48, 49) as shown in 2 meta-analyses (50, 51). However, most studies did not validate associations with mortality. Death in the ICU of 119 individuals was associated with changes in IL-6, IL-8, IL-10, VCAM-1, uPAR, IL-1RA, and TNF-α (52). Our data from the CORIMUNO SARI and TOCI trials (68% ward patients) confirmed associations between 90-day mortality and IL-6, IL-8, IL-10, VCAM-1, and TNF-α levels.

Both IL-6 and IL-10 were considered to derive the Dublin-Boston score (53). Such a score requires repeated assessment of the 4-day change in the untransformed IL-6/IL-10 ratio, which is not convenient and evaluates only the short-term evolution of patients. Unlike the CORIMUNO score, the 4-day change in the IL-6/IL-10 ratio was associated with a difference in short-term clinical outcome based on the clinical change from the day of study entry (day 0) to day 7, as measured using a 6-point ordinal scale endorsed by the WHO. In contrast, we found that both high IL-10 and, to a lesser extent, elevated IL-6 levels are associated with the risk of critical outcome. Although serum levels of IL-6, IL-10, and TNF-α are higher in males than in females (51), we found that including sex in our CORIMUNO score did not improve predictive power.

We have been surprised by the significant contribution of IL-10 levels to prognosis, as IL-10 is generally considered an antiinflammatory cytokine. Nonetheless, other, less well-known lines of evidence suggest that IL-10 may exhibit proinflammatory actions (54). For example, IL-10 administration in human experimental endotoxemia potentiated systemic levels of IFN-γ and its downstream chemokine target gene CXCL10 as well as CD8^+^ T cell and NK cell activity (55). Consistently, individuals with COVID-19 who displayed high serum levels of both IL-10 and IFN-γ also showed high CXCL10, granzyme A, and granzyme B.

We also confirmed that the fibrin degradation product D-dimer is elevated in severe COVID-19 (56, 57). In agreement with these reports, the elevation of D-dimers and vWF, as well as lower VEGF in fatal COVID-19 cases, provides evidence of endothelial injury in COVID-19, even in initially non-critically ill patients.

Neutrophilic inflammation may also contribute to endothelial injury. However, neutrophilia is predominantly a feature of the later phases of COVID-19. At the same time, markers of endothelial damage were increased in the first days of symptoms in our CORIMUNO cohorts, with high levels of P-selectin, E-selectin, L-selectin, endoglin, vWF-A2, sVCAM-1, and sICAM-1, and low VEGF serum levels at day 1. Consistently continued thrombotic events in late-stage fatal COVID-19 may result from neutrophil-mediated coagulation, as recently demonstrated in COVID-19 (58). Indeed, we observed elevated myeloperoxidase and IL-8 serum levels in our patients with COVID-19, with higher initial IL-8 levels and neutrophil counts in patients with a lethal outcome.

Early on, age was recognized as closely associated with COVID-19 severity (59, 60), as also observed in the CORIMUNO cohort. Older patients showed higher levels of inflammatory mediators even after multivariable severity adjustment, but IL-6 differences between survivors and non-survivors persisted after controlling for age, implying COVID-19–specific immune amplification.

Another salient finding of our study is the prognostic role of LCN2 and KIM-1 serum levels, which provided far more predictive information than eGFR, serum creatinine, and other biomarkers of AKI, such as cystatin C, RBP4, or TFF3. KIM-1 and LCN2 were elevated early, independent of renal failure, in patients with moderate and severe disease from an early stage of the infection. As reported by Vogel et al. (61), KIM-1 can detect AKI at an early stage and predict higher risk of ICU admissions among patients with COVID-19. The latest research, conducted in a small monocentric study, showed that the urine KIM-1/creatinine ratio was associated with COVID-19–specific mortality (62).

KIM-1 acts as a phosphatidylserine receptor to clear apoptotic cells. It is expressed in damaged kidney epithelial cells and confers phagocytic capacity (63–65) that protects the kidney (66). To validate the high immunoreactive KIM-1 abundance in TECs of kidney biopsies from patients with COVID-19, we reviewed publicly available scRNA-seq datasets of kidneys of patients with COVID-19. We found that among kidney cell populations, *HAVCR1* mRNA was expressed at the highest levels in kidney TECs, suggesting that TECs are the major cellular source of KIM-1 production in patients with COVID-19.

In contrast, LCN2 is distributed in many tissues (67–70). LCN2 is rapidly upregulated in ischemic kidney conditions (68). LCN2 may reflect not only COVID-19–induced kidney tubular injury but also neutrophil and macrophage activation and lung epithelial injury. Notably, it may amplify neutrophil-macrophage crosstalk and induce CXCR2, as shown in experimental nonalcoholic steatohepatitis (71). In contrast to KIM-1 immunostaining, which was widespread and consistently intense in COVID-19 kidney biopsies, LCN2 expression in the kidney was less consistently increased at both the protein and mRNA levels. Therefore, we suspect that LCN2 circulating levels may reflect inflammation or injury in other organs. IL-6–mediated hepatic LCN2 production is the primary source of plasma and urine LCN2 during experimental AKI (72). Given the very high levels of IL-6 in patients with COVID-19, especially those with adverse trajectories, we hypothesized that elevated LCN2 serum levels may reflect liver inflammation and the IL-6 response. Meanwhile, we determined LCN2 concentrations on day 6 during administration of sarilumab or tocilizumab and in matched individuals receiving usual care (data not shown). LCN2 levels (as well as other markers of AKI, such as KIM-1, osteopontin, RBP4, and TFF3) were unchanged after anti–IL-6R therapies during this timeframe, challenging the classical picture and implying IL-6–independent regulation.

Notably, a previous study reported that a substantial proportion of patients with COVID-19 exhibited AKI. Higher urinary KIM-1 and LCN2 were associated with the risk of a composite index, including stage 3 AKI, dialysis, and death (73). Our study differs from this previous one because of careful monitoring of pneumonia severity and because the time of blood collection was strictly controlled (within 72 hours of hospitalization, 11 days after symptom onset on average) as compared with urinary samples collected at a median of 9.5 (IQR, 4.5–17) days after hospital admission in patients who experienced the primary outcome, and at a median of 5 (IQR, 2–9) days after hospital admission in patients who did not experience the primary outcome, which may have contributed to significant bias in the former study (73).

Interestingly, the CORIMUNO score still distinguishes high-risk from low-risk patients in the anti–IL-6R and usual treatment groups, suggesting that these treatments did not affect key drivers of COVID-19 mortality. Meanwhile, IL-6R Abs were associated with a reduced risk of death, primarily in the high-risk category (by 20%), suggesting that the CORIMUNO score should help delineate patients most likely to benefit from such treatments. One limitation of our study is the lack of analysis of patients treated with glucocorticoids, a treatment that was not systematic at the time of the 2 trials. Nonetheless, given the overlapping actions of glucocorticoids with anti–IL-6R Ab, the predictive score may still be relevant.

Another limitation is that the derivation and validation cohorts included patients from the “first wave,” infected with A2/A3 and B/B.1/B1.1 lineages (74). Thus, the CORIMUNO score may be recalibrated for newer SARS-CoV-2 variants.

Solid epidemiological retrospective evidence indicated that survivors of COVID-19 after hospitalization were at higher risk of eGFR decline and end-stage kidney disease (75). An observational study showed that COVID-19–associated AKI was associated with higher mortality (76). Our study provides potentially novel insights by revealing the significant prognostic role of unrecognized acute kidney tubular injury in moderate to severe COVID-19 pneumonia, supporting early kidney monitoring and kidney-protective measures that may improve outcomes. Plasma KIM-1, a known predictor of end-stage kidney disease in diabetes (77) and chronic kidney disease progression in the healthy middle-aged population (78), was elevated even among survivors, suggesting long-term renal impact of COVID-19. Strikingly, the CORIMUNO score still achieved discrimination between the high-risk and low-risk of death groups, even in patients with normal eGFR on admission.

In conclusion, we demonstrated that a simple composite CORIMUNO score can be an integral component of a biologically based survival prediction model. This enables the collection of nearly complete survival information, which was previously unavailable with the proposed prediction models. In our evaluation of 196 patients with COVID-19 from 15 hospitals and 2 independent trials, the CORIMUNO score significantly (AUC = 0.83, 95% CI: 0.75−0.9) outperformed most laboratory tests and image-based visual and quantitative predictors in the prediction of disease progression and mortality and in the separation between the Kaplan-Meier survival curves of patients stratified into low- and high-risk groups (*P* < 10^–4^). We acknowledge that we do not know whether this CORIMUNO model could still be applied to the current patient population with COVID-19, but it is remarkable that the strong association of kidney-derived proteins with poor outcomes and mortality supports the notion that the kidney is an overlooked sentinel organ in SARS-CoV-2 pneumonia that should be considered in future studies of patients with apparently moderate forms of sepsis. Likewise, the higher levels of the classically antiinflammatory cytokine IL-10 in people at high risk of death is another little-explored paradox.

## Methods

### Sex as a biological variable.

Both sexes were included in this study, and sex was evaluated as a biological variable. No difference was observed between males and females, and data from both sexes are combined in the figures.

Patients’ recruitment in trials, the bead-based multianalyte Luminex multiplex assay, and the microfluidic cartridge–based immunoassay platform are described in the Supplemental Methods.

### Statistics.

The primary outcome of the prediction was death by day 90. The secondary outcome was an increase in the WHO CPS by more than 1 point by day 14. The proportion of patients with a WHO CPS (18) greater than 5 on the 10-point scale (moderate to critical) and survival without invasive or noninvasive ventilation at day 14 was monitored daily as previously described (18–20, 79). We also recorded death within the next 90 days. A statistical analysis plan is available in the supplemental materials.

The approach included multiplex profiling of 41 plasma proteins, plus albumin, D-dimer, creatinine, total lymphocyte, neutrophil, and platelet counts within 24 hours of trial randomization. All tabulated numerical data are summarized by the median and IQR for quantitative data and frequency and percentage on observed data for qualitative data, unless expressly stated. Comparisons were performed using the Wilcoxon rank-sum test for quantitative data and Fisher’s exact test for qualitative data. We next used regularization to control model complexity.

To overcome the risk of overfitting, we considered a multivariable model using a penalized logistic regression (LASSO) to predict death at 90 days. All variables with a *P* value below 0.05 in the univariate analyses were included in the model. The main interest of a LASSO regression is to handle overfitting, which is why we planned to apply this approach (80). The main idea of LASSO is to use a penalty term linked to model complexity to achieve the best trade-off between model complexity and the amount of information it carries. In practice, the penalization is modeled through a parameter (lambda). Figure 4 shows the model’s complexity as a function of lambda. Optimization of this parameter could be obtained by cross-validation (81). Final associations were estimated using odds ratios and 95% CI. Then, ROC curves were generated from the linear predictor of the previous model, and the AUCs were calculated to evaluate the model’s performance in the training, validation, and combined cohorts. We compared global discrimination by calculating the AUC for each score, with 95% CI estimated by the DeLong method, and tested differences in AUCs using the nonparametric DeLong test for correlated ROC curves. We calculated the corresponding *P* values from the DeLong test, comparing each component with the composite score (82). The risk threshold was determined with the Youden index.

Survival rates and 95% CIs were estimated using the Kaplan-Meier estimator and compared using log-rank tests. All tests are 2-sided and a *P* value less than 0.05 was the cutoff used to determine significance. All statistical analyses were performed using the open-source software R (version 4.1.0; https://CRAN.R-project.org/package=survival). The main packages used were survival, pROC (83), and glmnet (81).

Hierarchical clustering of standardized biomarker concentrations was performed using Euclidean distance and Ward’s minimum variance method to explore similarities among biomarkers. Relationships between biomarkers were further examined using Spearman’s correlation coefficients, displayed as a color-coded correlation matrix to illustrate the strength and direction of associations. Principal component analysis was also performed to explore overall patterns and relationships among biomarkers.

### Study approval.

The trials were approved nationally by the ethics committee on March 23, 2020 (file #20.03.20.56342, CPP Ile-de-France VI, EudraCT: 2020-001246-18), and by the French Medicines Agency, ANSM, 143 boulevard Anatole-France, 93285 Saint-Denis Cedex, France, and the Commission Nationale de l’Informatique et des Libertés (CNIL), Paris, France. Written informed consent was obtained from all patients or from the patient’s legal representative if the patient was too unwell to provide consent to enter the CORIMUNO cohort.

For the COVID-19 autopsy cohort, the first sample of patient tissues was obtained from autopsies performed at the Institute of Legal Medicine of the University Medical Center Hamburg-Eppendorf, as previously published (84). From every liver and kidney specimen collected by the Institute of Legal Medicine, multiple randomly chosen small samples were available for different analyses. The Hamburg Chamber of Physicians’ ethics committee was informed of the study (2020-10353-BO-ff and PV7311). Controls included cases of sudden noninfectious deaths. The average postmortem interval was 6 days. Written informed consent was obtained from a legal representative or next of kin for autopsy and tissue sampling. No compensation was paid. The study protocol for clinical data collection (patient cohorts) was approved by the IRB of the University of Michigan (HUM00178971) and the University Medical Center Hamburg-Eppendorf. The IRB approved a waiver of informed consent for this observational study.

### Data availability.

Supporting data values are available in the supplemental material. The protocol, consent form, statistical analysis plan, regulatory documents, and other relevant study materials are available. As described in the protocol, the trial steering committee will facilitate access to the study data, and approval will not be unreasonably withheld. Deidentified participant data collected during the CORIMUNO-SARI and the CORIMUNO-TOCI trials (and the data dictionary) will be made available to bona fide researchers registered with an appropriate institution within 3 months of publication and for 10 years. Proposals should be addressed via email to raphael.porcher@aphp.fr and will be reviewed by the CORIMUNO-19 scientific committee. The steering committee will need to be satisfied that any proposed publication is of high quality, honors the commitments made to the study participants in the consent documentation and ethical approvals, and is compliant with relevant legal and regulatory requirements (e.g., data protection and privacy). To gain access, data requesters will need to sign a data access agreement and confirm that the data will be used only for the agreed-upon purpose for which access was granted. The steering committee will have the right to review and comment on any draft manuscripts before publication.

## Author contributions

PLT, OH, XM, PR, and MRR conceived the project and designed the experiments. OL, F Morin, LR, NM, VGP, NW, JD, and TIG performed most of the experiments, with assistance from all authors. NW, JB, and BI performed bioinformatics analyses. AWP, RP, and MRR performed statistical analyzes. SF, MZ, ZAM, ME, AC, OM, XB, DB, IB, SB, HI, HF, AM, VA, AS, ED, GH, ILMDG, CM, LH, CV, ST, OK, VP, MD, ML, AK, and SCZ collected and analyzed the biological samples and clinical data with the CORIMUNO-19 collaborative group. OL, F Morin, SCZ, YY, RP, PR, XM, OH, MRR, and PLT provided scientific input and contributed to discussions. OL, F. Morin, AWP, and LR share first authorship. The order of the first authors was determined based on their respective laboratories’ investment in the project. Because OL, and LR belong to the same laboratory, LR was placed fourth. The CORIMUNO-19 collaborative group writing committee members are PLT, OL, AWP, MRR, XM, OH, RP, and PR. The steering committee members are PR (chair of the CORIMUNO-19 platform), SB, MD, OH, XM, MRR, PLT, and AT. BT, YL, MJ, TBH, JSH, CL, JG, F Mentré enrolled patients or collected biological samples.

## Funding support

French Ministry of Health, PHRC (PHRC COVID-19–20–0151, PHRC COVID-19–20–0029).

- Fondation de l’Assistance Publique Hôpitaux de Paris (Alliance Tous Unis Contre le Virus).
- FRM grant (REA202010012514) to PLT.
- ANRS grant (ANRS0147) via VINTED sponsorship to PLT.
- DFG (CRC 1648) to TBH.
- European Research Council (CureFSGS, project 101141768) to TBH.
- Horizon Europe Framework Programme (project 101095146-Prime-CKD) to TBH.

## Supplementary Material

Supplemental data

ICMJE disclosure forms

Supplemental data set 1

Supplemental data set 2

Supporting data values

## Figures and Tables

**Table 1:**
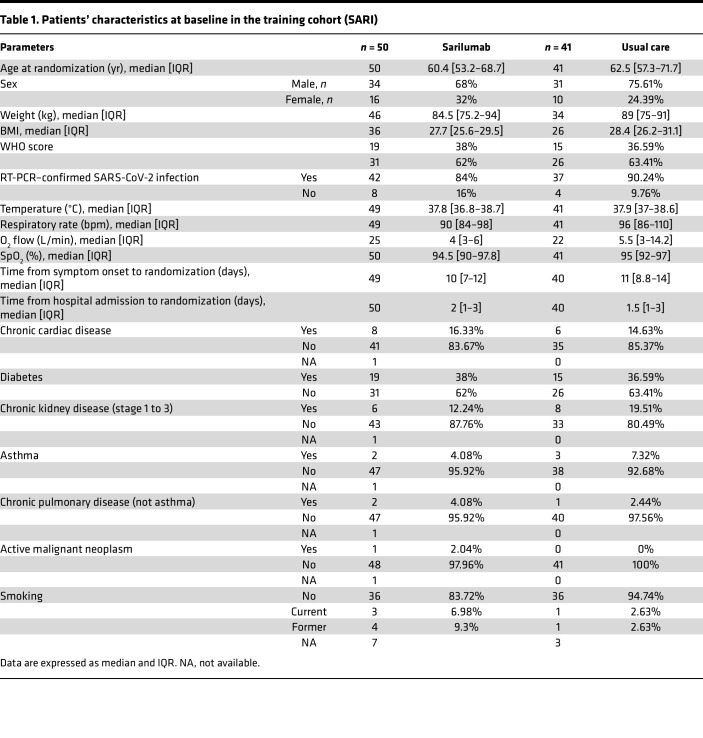
Patients’ characteristics at baseline in the training cohort (SARI)

**Table 2:**
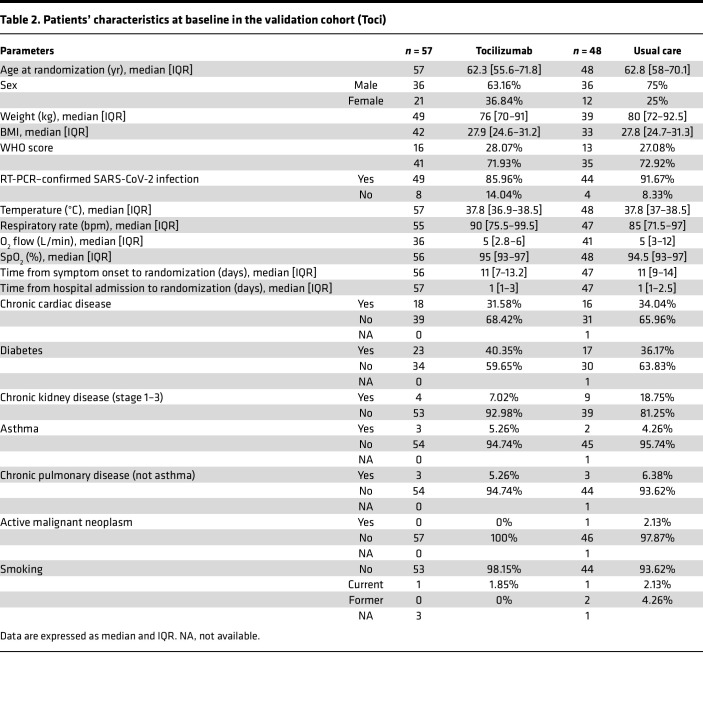
Patients’ characteristics at baseline in the validation cohort (Toci)

**Table 3:**
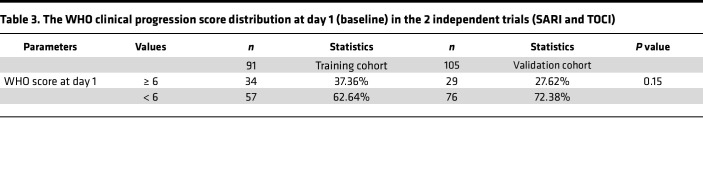
The WHO clinical progression score distribution at day 1 (baseline) in the 2 independent trials (SARI and TOCI)

**Table 4:**
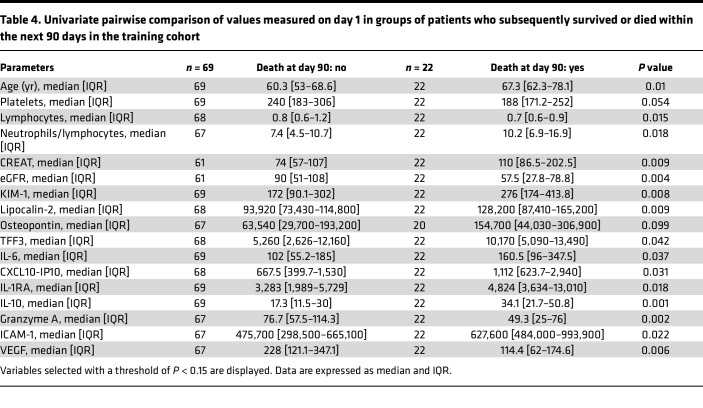
Univariate pairwise comparison of values measured on day 1 in groups of patients who subsequently survived or died within the next 90 days in the training cohort

**Table 5:**
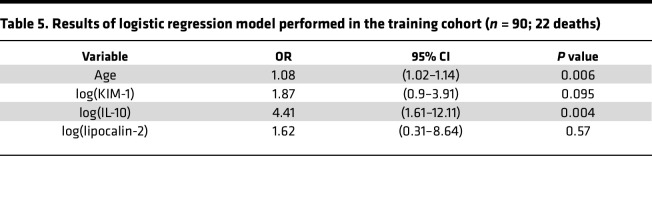
Results of logistic regression model performed in the training cohort (*n* = 90; 22 deaths)

**Table 6:**
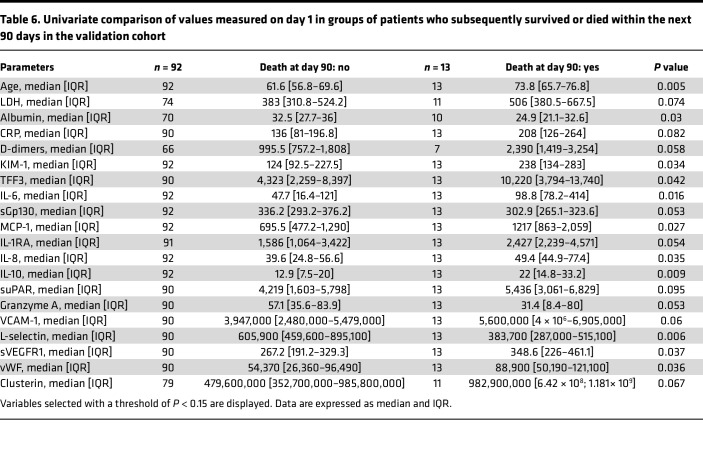
Univariate comparison of values measured on day 1 in groups of patients who subsequently survived or died within the next 90 days in the validation cohort

**Table 7:**
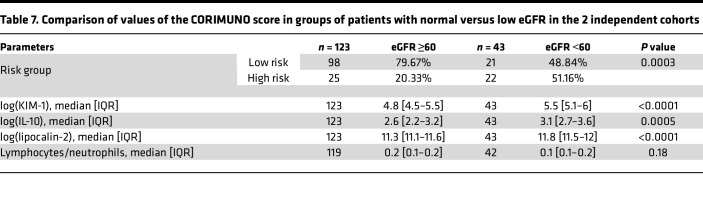
Comparison of values of the CORIMUNO score in groups of patients with normal versus low eGFR in the 2 independent cohorts

## Version 1 01/23/2026

Electronic publication
