# Supplementary material for: Levels of circulating kidney injury markers and IL-10 identify non-critically ill patients with COVID-19 at risk of death
Source: JCI Insight. 2026 Jan 23;11(2):e198244. doi: 10.1172/jci.insight.198244 (PMC12892917; doi:10.1172/jci.insight.198244)
Supplement: Supplemental data [file jciinsight-11-198244-s307.pdf]

**Supplemental Methods:**

**Cohort of patients with COVID-19-associated nephropathy**

We conducted an immunofluorescence study on anonymized kidney biopsies from a multicenter, retrospective case series of patients with COVID-19 who developed acute kidney injury (AKI) and/or proteinuria and underwent a kidney biopsy in Paris and its metropolitan area. All clinical investigations have been conducted in accordance with the principles of the Declaration of Helsinki.

Thirty-one adult patients who tested positive for SARS-CoV-2 by polymerase chain reaction on a nasopharyngeal swab or a pulmonary sample were hospitalized in five Nephrology departments in the Paris metropolitan area from March 2020 to June 2021, and were eligible. Patients with a history of end-stage kidney disease were excluded. This study was conducted in compliance with the Helsinki Declaration and approved by the local institutional review board as minimal-risk research, utilizing retrospective data collected for routine clinical practice. Due to the study's observational nature, patient consent was waived per French law.

**Immunofluorescence staining**

Kidney biopsy specimens with sufficient tissue for immunohistochemical evaluation, following completion of the diagnostic workup, were included. Dual KIM-1/ACE2 and LCN2/KIM-1 immunostaining could be performed in 37 and 17 COVID-19 kidney samples, respectively, and in 8 and 5 non-COVID-19 controls. For immunofluorescence, paraffin-embedded sections were stained with the following primary antibodies: rabbit anti-ACE2 (Bio-techne &D systems NBP2-67692, 1:150), goat anti-KIM-1 (Bio-techne &D systems AF1750, 1:200), rabbit anti-LCN2 (Atlas antibodies HPA002695, 1:500). The following secondary antibodies were used: Donkey Anti-Rabbit IgG (H+L) cya3 (Jackson Immunoresearch 711-165-152, 1:250, Donkey Anti-Goat IgG (H+L) Alexa Fluor® 647 (Jackson Immunoresearch 705-605-147, 1:250). The nuclei were stained with Hoechst (33342). Whole images

were taken with an Olympus SLIDEVIEW VS200 slide scanner. Quantification was done using the HALO® image analysis software (Indica Labs, Albuquerque, USA).

**RNA expression analyses in tissues from deceased patients with critical COVID-19**

For the liver, n = 7 controls (non-COVID autopsy) and n = 11 COVID cases (patients with PCR-positive results in the lung) were selected. For the kidney, n = 10 controls and n = 20 COVID cases were selected. Data from some of the liver specimens have already been reported (1). RNA was extracted using QIAGEN RNeasy® Micro kit according to the manufacturer's instructions, followed by Agilent Bioanalyzer sample quality control, library preparation with Lexogen Corall Total RNA, and rRNA depletion. Single-end RNA sequencing was performed on a 75-bp NextSeq v2.5, yielding more than 30 million reads. Raw reads were trimmed using TrimGalore! V0.4.3 (http://www.bioinformatics.babraham.ac.uk/projects/trim_galore/) and aligned using RNA STAR (2). Mapped reads were counted with HTSeq (3). Normalized read counts were obtained using DESeq2-normalized counts (4). For every graph, the ROUT outlier detection algorithm with Q = 1% was run using GraphPad Prism 9, which removed 0-2 samples per dataset. U Mann-Whitney tests were performed within individual comparisons for each gene and organ, respectively. Only statistically significant differences are shown in the graph.

**Single-nuclei RNA sequencing (snRNA-seq) data analysis**

To delineate the cellular origin of markers of interest, the study was complemented with data mining from a single-cell atlas of an autopsy cohort comprising 20 male and 12 female donors of various ages (ranging from 30 to 89 years), as previously published (5). A biobank was created with a subset of 17 donors. We collected tissue from the lungs, kidneys, hearts, and livers of most donors, preserving specimens for single-cell and spatial analysis as previously reported (5). Data were obtained from the Single Cell Portal (SCP) (5, 6). Dot plots for kidney samples were generated in Python v3.9.6using

scanpy v1.9.3 [Ref https://genomebiology.biomedcentral.com/articles/10.1186/s13059-017-1382-0]. Dot plots for lung samples were generated in R version 4.2.1 using Seurat version 4.3.0 (7).

**Patient recruitment in trials**

**Training cohort**: To test these hypotheses, we performed a multiplexed immunoassay-based study on 196 patients enrolled in two multicenter, open-label, randomized, Phase 2/3 clinical trials nested within the CORIMUNO-19 cohort (Cohort of Multiple Randomized Controlled Trials Open-label of Immune Modulatory Drugs and Other Treatments in COVID-19 Patients), NCT04324047. These two trials were conducted in March and April 2020. First, we used data from the CORIMUNO-SARI multicenter, adaptive, open-label study, which enrolled patients with COVID-19 from six French hospitals in randomized controlled trials of different therapeutic regimens (the CORIMUNO-19 cohort). Patients with moderate-to-severe pneumonia were enrolled in the CORIMUNO-SARI-1 trial (8). These analyzed patients were 18 years or older, hospitalized with COVID-19 in 6 French centers, requiring at least 3L/min of oxygen either without ventilation assistance and a WHO Clinical Progression Scale [CPS] score of 5 or with high-flow or with mechanical ventilation assistance (CPS $\geq$ 6) (9). This completed trial is closed to new participants and is registered on ClinicalTrials.gov under NCT04324073.

Measurements of the 42 biological parameters were available in 91 subjects. Their clinical characteristics are summarized in Tables 1 and 3.

**Validation cohort**: Next, we replicated these measurements for external validation using an independent study population of 105 subjects enrolled in the CORIMUNO-TOCI trial, a multicenter randomized clinical trial involving 9 hospitals, with the same inclusion criteria as previously described (10, 11). This completed trial is now closed to new participants and is registered with ClinicalTrials.gov under the identifier NCT04331808. The clinical characteristics of patients are summarized in Tables 2 and 3.

**Bead-based multianalyte Luminex multiplex assay**

The serum concentrations of inflammatory mediators (the interleukin-6 system (soluble Interleukin 6 RA (sIL6RA), soluble gp130), the interleukin-1 system (interleukin 1 alpha (IL1$\alpha$), interleukin 1 beta (IL1$\beta$)), interleukin 17A (IL17A), interferon-gamma (IFN$\gamma$), C-X-C motif chemokine ligand 10 (CXCL10)/Interferon gamma-induced protein 10 (IP-10), granzyme A and granzyme B), were complemented by molecules involved in endothelial maintenance (Vascular Endothelial Growth Factor A (VEGFA), soluble VEGF Receptor 1 (sVEGFR1), Platelet-derived Growth Factor AA (PDGF-AA), PDGF-BB, soluble endoglin (sEng), Placental Growth Factor (PlGF), Basic Fibroblast Growth Factor (bFGF)), and of vascular injury (soluble E-selectin, soluble P-selectin, soluble L-selectin, soluble urokinase plasminogen activator (sUPAR), soluble InterCellular Adhesion Molecule 1 (sICAM-1), soluble Vascular Cell Adhesion Molecule 1 (sVCAM-1), soluble von Willebrand Factor (vWF), Clusterin). We combined the above-cited analytes with markers of kidney dysfunction (estimated glomerular filtration rate (eGFR) and injury: serum concentrations of Cystatin C, Retinol Binding protein 4 (RBP4), Lipocalin 2 (LCN2) (also named as neutrophil gelatinase-associated lipocalin (NGAL)), Osteopontin, and Trefoil Factor 3 (TFF3). These molecules were measured using Biotechne Luminex High-Performance assay kits according to manufacturer recommendations. Serum samples were thawed and centrifuged at 16,000 g for 4 min immediately before use. Reads and calculations of results were done on a Bio-Rad Bio-Plex station. Three persons carried out all these measurements, centralized at the Paris Cardiovascular Center (PARCC), Inserm, Paris.

**Microfluidic cartridge-based immunoassay platform**

We assessed serum concentration of IL-6 (using 2nd generation kits), IL-8, IL-10, CCL2/monocyte chemoattractant protein-1 (MCP-1), interleukin 1 RA (IL1RA), tumor necrosis factor-alpha (TNF$\alpha$), and kidney injury molecule-1 (KIM-1)/HAVCR, in a centralized manner using an Ella® platform

(ProteinSimple & Biotechne) which is routinely and widely used for the quantification of soluble biomarkers at the Immunology and Histocompatibility Laboratory, Saint-Louis hospital, AP-HP, Paris.

**Supplemental references:**


1. Wanner N, Andrieux G, Badia IMP, Edler C, Pfefferle S, Lindenmeyer MT, et al. Molecular consequences of SARS-CoV-2 liver tropism. *Nat Metab.* 2022;4(3):310–9.
2. Dobin A, Davis CA, Schlesinger F, Drenkow J, Zaleski C, Jha S, et al. STAR: ultrafast universal RNA-seq aligner. *Bioinformatics.* 2013;29(1):15–21.
3. Anders S, Pyl PT, and Huber W. HTSeq--a Python framework to work with high-throughput sequencing data. *Bioinformatics.* 2015;31(2):166–9.
4. Love MI, Huber W, and Anders S. Moderated estimation of fold change and dispersion for RNA-seq data with DESeq2. *Genome biology.* 2014;15(12):550.
5. Delorey TM, Ziegler CGK, Heimberg G, Normand R, Yang Y, Segerstolpe A, et al. COVID-19 tissue atlases reveal SARS-CoV-2 pathology and cellular targets. *Nature.* 2021;595(7865):107–13.
6. Melms JC, Biermann J, Huang H, Wang Y, Nair A, Tagore S, et al. A molecular single-cell lung atlas of lethal COVID-19. *Nature.* 2021;595(7865):114–9.
7. Hao Y, Hao S, Andersen-Nissen E, Mauck WM, 3rd, Zheng S, Butler A, et al. Integrated analysis of multimodal single-cell data. *Cell.* 2021;184(13):3573–87 e29.
8. group C-C. Sarilumab in adults hospitalised with moderate-to-severe COVID-19 pneumonia (CORIMUNO-SARI-1): An open-label randomised controlled trial. *Lancet Rheumatol.* 2022;4(1):e24–e32.
9. Characterisation WHOWGotC, and Management of C-i. A minimal common outcome measure set for COVID-19 clinical research. *Lancet Infect Dis.* 2020;20(8):e192–e7.
10. Mariette X, Hermine O, Tharaux PL, Resche-Rigon M, Steg PG, Porcher R, et al. Effectiveness of Tocilizumab in Patients Hospitalized With COVID-19: A Follow-up of the CORIMUNO-TOCI-1 Randomized Clinical Trial. *JAMA internal medicine.* 2021;181(9):1241–3.

11. Hermine O, Mariette X, Tharaux PL, Resche-Rigon M, Porcher R, Ravaud P, et al. Effect of Tocilizumab vs Usual Care in Adults Hospitalized With COVID-19 and Moderate or Severe Pneumonia: A Randomized Clinical Trial. *JAMA internal medicine.* 2021;181(1):32–40.

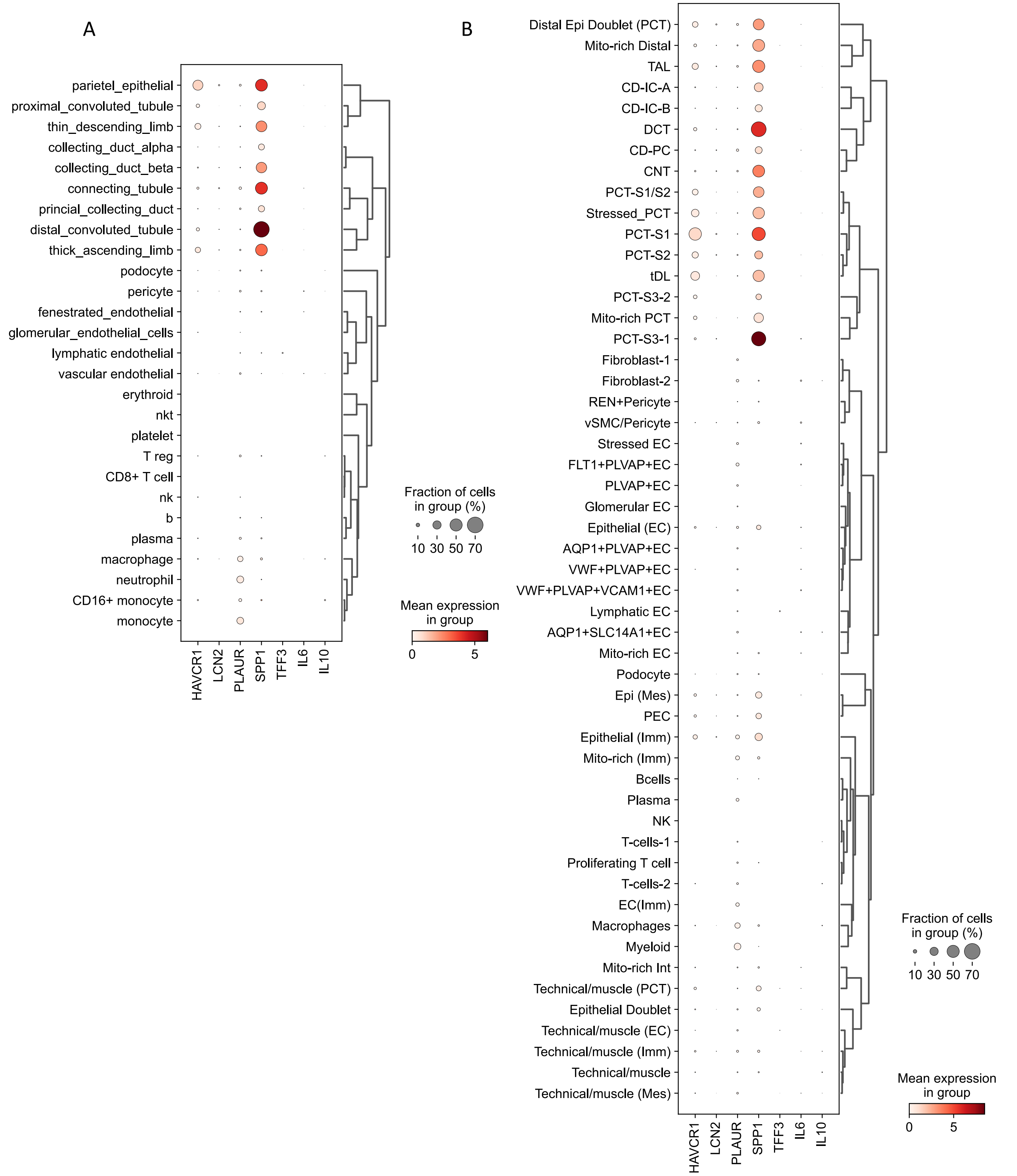


**Supplemental Figure 1**: **A**. Relative abundance of HAVCR1, LCN2, PLAUR, SPP1, TFF3, IL6 and IL10 mRNA in kidney cell types. Pooled results from single-cell COVID-19 atlas from 16 post-mortem patients deceased from COVID-19 critical pneumonia. **B**. Relative abundance of HAVCR1, LCN2, PLAUR, SPP1, TFF3, IL6 and IL10 mRNA in detailed subsets of kidney cells. CD: collecting duct; IC: intercalated cells; DCT: distal convoluted tubules; EC: endothelial cells; Mes: mesangial cells; PEC: parietal epithelial cells; PCT: proximal convoluted tubules; PCT-S1: proximal convoluted tubules segment 1; PCT-S2: proximal convoluted tubules segment 2; TAL: thick ascending limb; tDL: thin descending limb; NK: natural killer cells; NKT: natural killer T cells; T reg: regulatory T lymphocytes; T cell: T ymphocytes; B: B lymphocytes; plasma: plasma cells; vSMC: vascular smooth muscle cells.

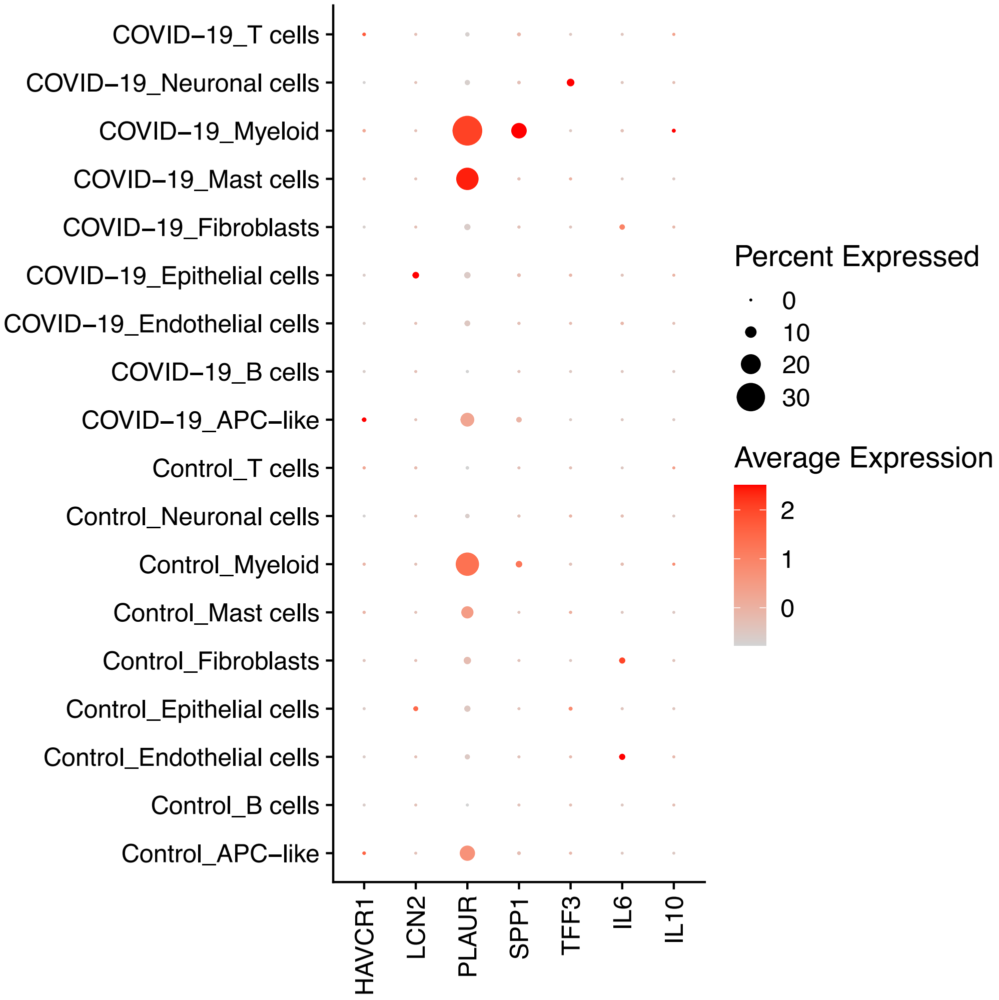


**Supplemental Figure 2**: Relative abundance of HAVCR1, LCN2, PLAUR, SPP1, TFF3, IL6 and IL10 mRNA in lung cell types. Pooled results from single-cell COVID-19 atlas from16 post-mortem patients deceased from COVID-19 critical pneumonia.

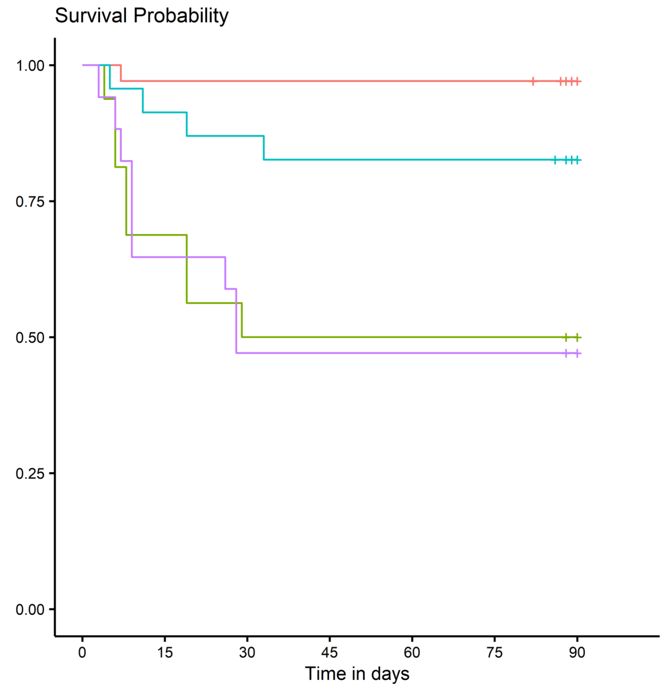


No. at risk

| | 0 | 15 | 30 | 45 | 60 | 75 | 90 |
|---|---|---|---|---|---|---|---|
| Sari / Low risk | 34 | 33 | 33 | 33 | 33 | 33 | 23 |
| Sari / High risk | 16 | 11 | 8 | 8 | 8 | 8 | 7 |
| Usual care / Low risk | 23 | 21 | 20 | 19 | 19 | 19 | 15 |
| Usual care / High risk | 17 | 11 | 8 | 8 | 8 | 8 | 7 |

**Supplemental Figure 3**: Kaplan-Meier survival curves for COVID-19 patients stratified by risk group (high/low) and treatment arm (Sarilumab/Usual Care) in the training cohort.

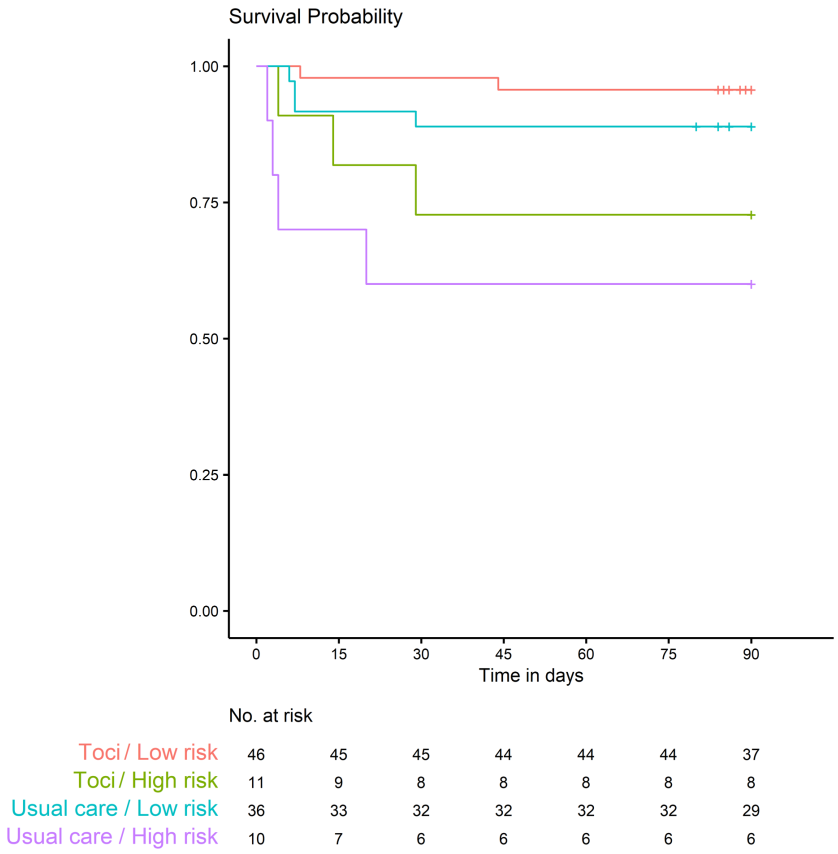


**Supplemental Figure 4**: Kaplan-Meier survival curves for COVID-19 patients stratified by risk group (high/low) and treatment arm (Tocilizumab/Usual Care) in the validation cohort.

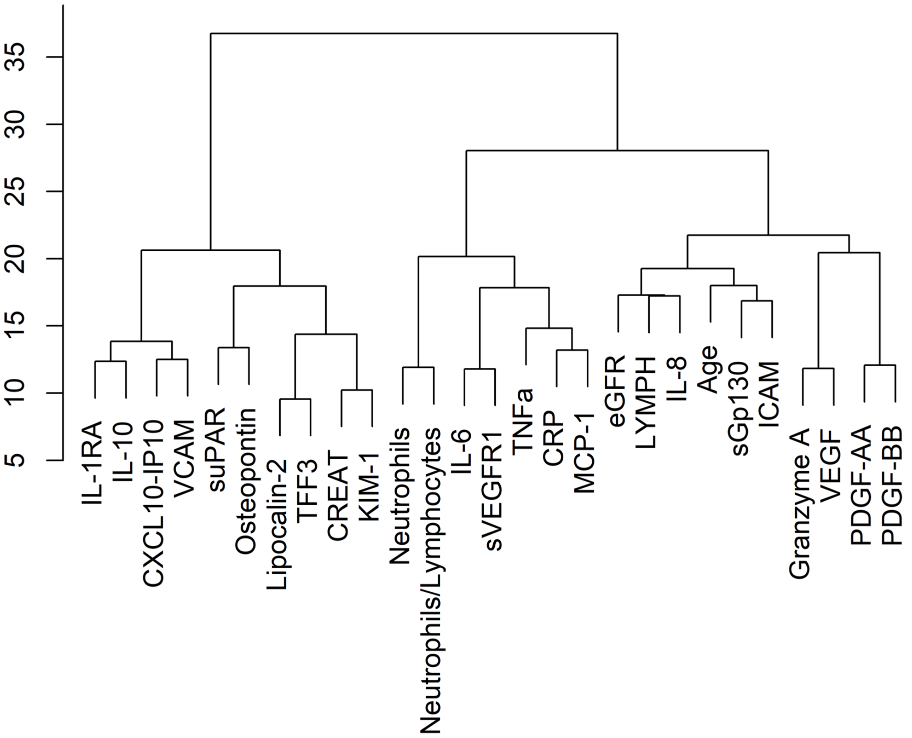


**Supplemental Figure 5**: Cluster dendrogram of biomarker concentrations in the pooled cohorts. The serum biomarker concentrations were analyzed by unsupervised clustering to determine how similar the values were to each other. The unsupervised clustering resulted in groupings similar to functional groupings for some of the biomarkers. For instance, many of the proteins in the group associated with soluble forms of the interleukin-6 (IL-6) and VEGF receptor 1 (VEGFR1) were clustered in the same group that was distinct from the group containing anti-inflammatory IL-10 and IL-1 receptor antagonist (IL-1Ra). Lipocalin-2 (LCN2), TFF3 and kidney injury molecule-1 (KIM-1) were clustered in the same group. Although C-reactive protein (CRP) levels were classically correlated with IL-6 and MCP-1 levels as well as neutrophils count (NEUTRO), they were less discriminating the group of non-survivors from the group of survivors.

| | LYMPH | Neutrophils | CREAT | eGFR | sGp130 | KIM-1 | MCP-1 | TNFa | CXCL10-IP10 | IL-1RA | IL-6 | IL-8 | IL-10 | suPAR | ICAM | VCAM | sVEGFR1 | Granzyme A | VEGF | PDGF-AA | PDGF-BB | Lipocalin-2 | Osteopontin | TFF3 | Age | Neutrophils/Lymphocytes |
|---|---|---|---|---|---|---|---|---|---|---|---|---|---|---|---|---|---|---|---|---|---|---|---|---|---|---|
| Age | | | | | | | | | | | | | | | | | | | | | | | | | | 0.2 |
| TFF3 | | | | | | | | | | | | | | | | | | | | | | | | | 0.3 | 0.2 |
| Osteopontin | | | | | | | | | | | | | | | | | | | | | | | | 0.5 | 0 | 0.2 |
| Lipocalin-2 | | | | | | | | | | | | | | | | | | | | | | | 0.5 | 0.7 | 0.1 | 0.2 |
| PDGF-BB | | | | | | | | | | | | | | | | | | | | | | 0.1 | 0.1 | 0.2 | 0 | 0 |
| PDGF-AA | | | | | | | | | | | | | | | | | | | | | 0.8 | 0 | 0 | 0.1 | -0.1 | -0.1 |
| VEGF | | | | | | | | | | | | | | | | | | | | 0.3 | 0.2 | -0.1 | -0.2 | -0.1 | -0.1 | -0.1 |
| Granzyme A | | | | | | | | | | | | | | | | | | | 0.5 | 0.1 | 0 | 0 | -0.1 | 0.1 | -0.2 | -0.1 |
| sVEGFR1 | | | | | | | | | | | | | | | | | | 0.1 | 0.1 | 0.1 | 0 | 0.2 | 0 | 0.2 | 0.1 | 0.1 |
| VCAM | | | | | | | | | | | | | | | | | 0.3 | 0 | 0 | -0.1 | 0 | 0.3 | 0.4 | 0.4 | 0.2 | 0.2 |
| ICAM | | | | | | | | | | | | | | | | 0.1 | 0.2 | -0.2 | 0 | 0.1 | 0.1 | 0.1 | 0.1 | 0 | 0.1 | 0.1 |
| suPAR | | | | | | | | | | | | | | | 0.4 | 0.4 | 0.4 | -0.1 | 0.2 | 0.1 | 0.1 | 0.2 | 0.3 | 0.2 | 0.1 | 0.2 |
| IL-10 | | | | | | | | | | | | | | 0.2 | 0 | 0.5 | 0.2 | 0.1 | -0.2 | -0.2 | -0.2 | 0.3 | 0.3 | 0.3 | 0 | 0.2 |
| IL-8 | | | | | | | | | | | | | 0.3 | 0.2 | 0 | 0.1 | 0.2 | 0.2 | 0.1 | -0.1 | -0.2 | 0 | 0 | 0 | 0 | 0.2 |
| IL-6 | | | | | | | | | | | | 0.5 | 0.5 | 0.3 | 0.1 | 0.3 | 0.2 | 0.1 | -0.1 | -0.2 | -0.1 | 0.2 | 0.3 | 0.2 | 0.1 | 0.4 |
| IL-1RA | | | | | | | | | | | 0.6 | 0.4 | 0.7 | 0.3 | 0 | 0.5 | 0.3 | 0.2 | -0.1 | -0.2 | -0.2 | 0.3 | 0.3 | 0.3 | 0 | 0.3 |
| CXCL10-IP10 | | | | | | | | | | 0.5 | 0.4 | 0.2 | 0.7 | 0.1 | -0.1 | 0.5 | 0 | -0.1 | -0.4 | -0.3 | -0.2 | 0.4 | 0.5 | 0.5 | 0.2 | 0.3 |
| TNFa | | | | | | | | | 0.3 | 0.5 | 0.4 | 0.4 | 0.3 | 0.1 | 0 | 0.2 | 0.3 | 0.3 | 0.1 | -0.1 | -0.1 | 0.4 | 0.1 | 0.4 | 0.1 | 0.3 |
| MCP-1 | | | | | | | | 0.5 | 0.4 | 0.5 | 0.5 | 0.4 | 0.5 | 0.2 | 0 | 0.2 | 0.2 | 0.3 | 0 | -0.1 | -0.1 | 0.3 | 0.2 | 0.2 | 0.1 | 0.3 |
| KIM-1 | | | | | | | 0.2 | 0.4 | 0.3 | 0.4 | 0.4 | 0.3 | 0.3 | 0.2 | 0.2 | 0.3 | 0.2 | 0.1 | -0.1 | -0.1 | -0.1 | 0.4 | 0.2 | 0.5 | 0.3 | 0.2 |
| sGp130 | | | | | | 0.1 | -0.1 | -0.1 | -0.1 | -0.1 | -0.2 | 0 | 0.1 | 0 | 0 | 0.1 | 0 | 0 | 0 | 0.1 | 0.1 | 0 | 0 | 0 | -0.1 | -0.1 |
| eGFR | | | | | -0.1 | -0.3 | -0.1 | -0.4 | -0.3 | -0.3 | -0.1 | -0.1 | -0.4 | -0.2 | 0 | -0.4 | -0.2 | 0 | 0.1 | 0.1 | 0.1 | -0.4 | -0.1 | -0.5 | -0.3 | -0.1 |
| CREAT | | | | -0.9 | 0 | 0.3 | 0.2 | 0.4 | 0.4 | 0.4 | 0.1 | 0.1 | 0.4 | 0.3 | 0 | 0.4 | 0.3 | 0 | 0 | -0.1 | -0.1 | 0.5 | 0.1 | 0.5 | 0.1 | 0.1 |
| Neutrophils | | | 0.1 | -0.1 | -0.2 | 0.1 | 0.3 | 0.3 | 0.1 | 0.4 | 0.4 | 0.2 | 0.2 | 0.1 | 0 | 0.1 | 0.2 | 0.1 | 0.1 | 0.1 | 0 | 0.1 | 0 | 0.1 | 0 | 0.6 |
| LYMPH | | 0.2 | -0.1 | 0.1 | 0.1 | -0.2 | -0.1 | -0.1 | -0.2 | -0.1 | -0.2 | -0.1 | -0.1 | -0.1 | -0.2 | -0.2 | 0 | 0.2 | 0.1 | 0.2 | 0.1 | -0.2 | -0.2 | -0.2 | -0.2 | -0.6 |
| CRP | -0.1 | 0.4 | 0.1 | 0 | -0.3 | 0.2 | 0.4 | 0.3 | 0.3 | 0.3 | 0.4 | 0.2 | 0.2 | 0.2 | 0 | 0.1 | 0.1 | 0 | 0 | -0.1 | -0.1 | 0.2 | 0.2 | 0.1 | 0.1 | 0.4 |

[-1,-0.67]
(-0.67,-0.33]
(-0.33,0]
(0,0.33]
(0.33,0.67]
(0.67,1]

**Supplemental Figure 6**: Correlation matrix table showing correlation coefficients between variables that are associated with risk of death with 6 classes of color-coding of the values according to the correlation statistics.

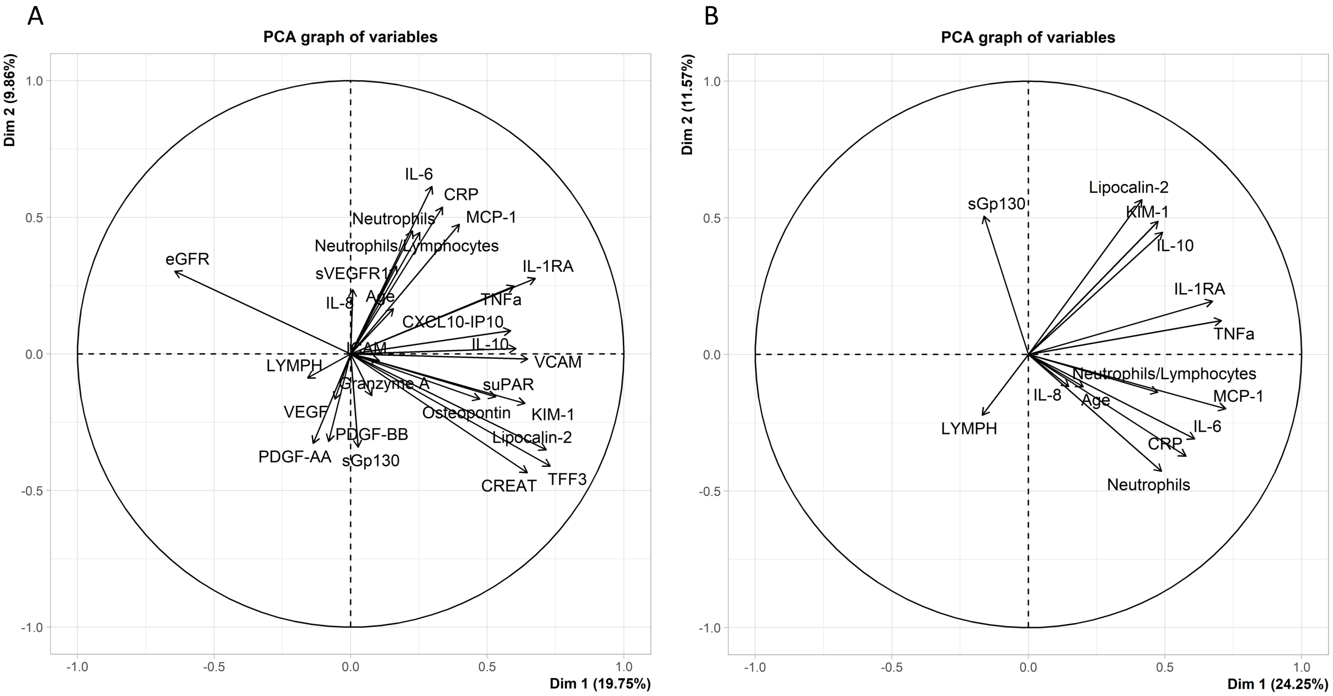


**Supplemental Figure 7**: **A**. PCA score plot of the first two PCs of data set about profiles of biomarkers independently associated with death in patients with some missing measurements (n=157 subjects, 29 (18.47%) dead by day 90). The figure below displays the relationships between all 26 variables simultaneously (assuming creatinine (CREAT) and eGFR represent the same thing). The first component explains 19.76 % of the variation, and the second component 10.32 %. The further away from the plot origin a variable lies, the stronger the impact that variable has on the model.
**B**. PCA score plot of the first two PCs of data set about biomarkers independently associated with death in 183 patients (34 (18.58%) dead by day 90).

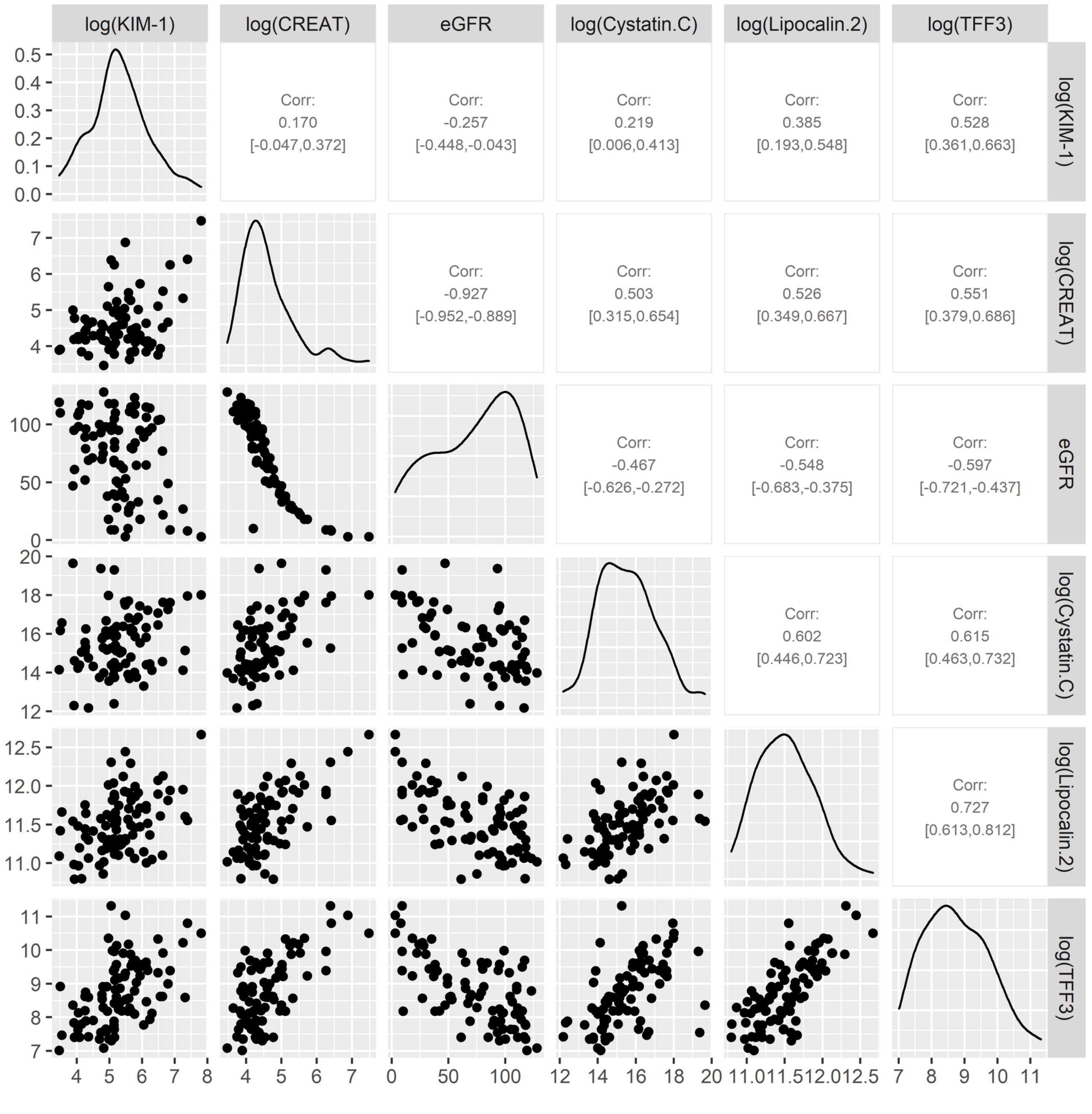


**Supplemental Figure 8**: distribution curves and correlation coefficient for the variables linked to kidney injury (KIM-1 log, Creatininemia log, eGFR, Cystatin c log, LCN2 log and TFF3 log). Data are presented as Spearman correlation coefficient (r) with 95% CI.

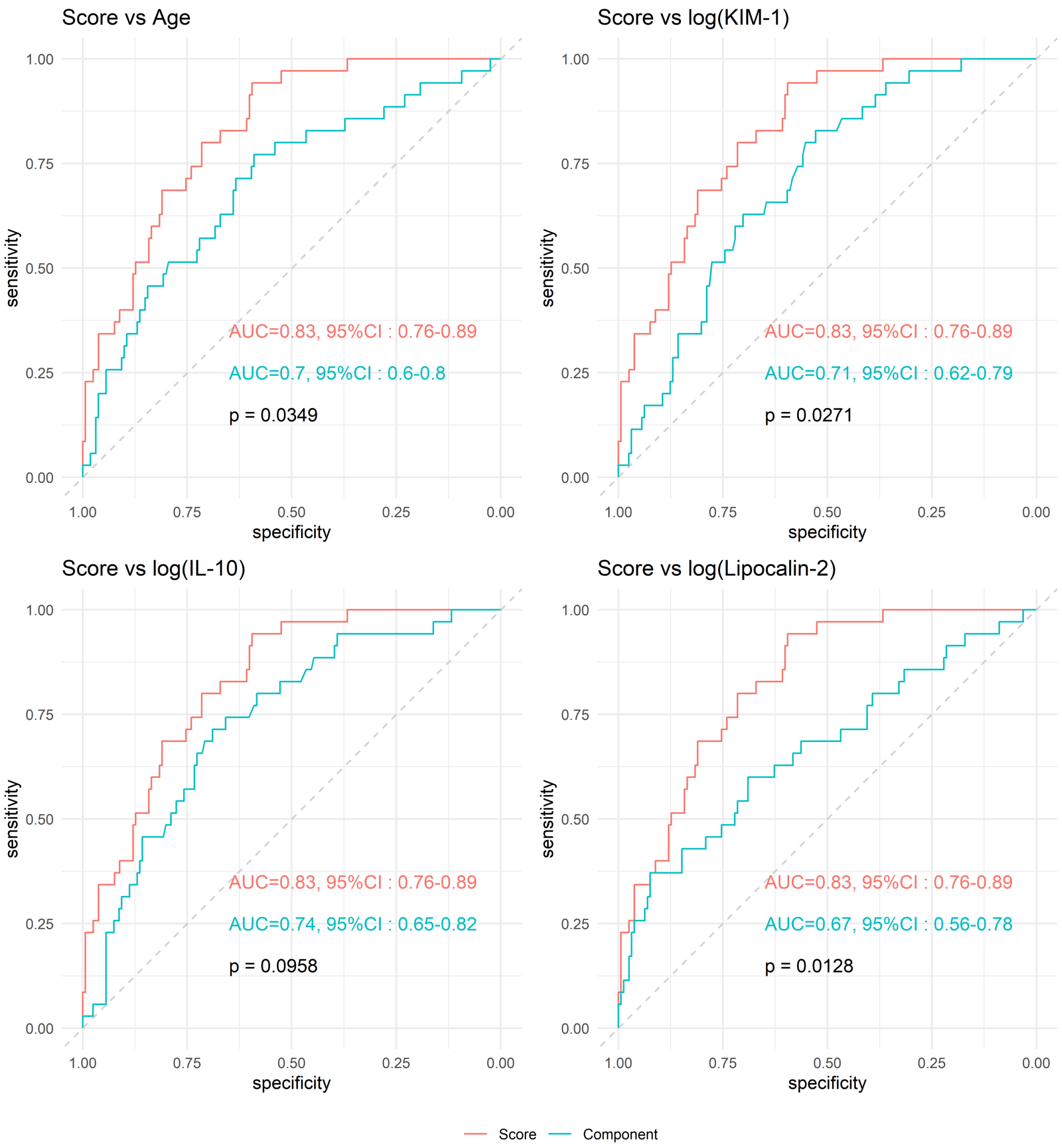


**Supplemental Figure 9**: Comparison of the respective ROC curves and AUC for each variable component of the global CORIMUNO SCORE (age, KIM-1, IL-10, LCN2)(cyan curve), along with the overall score itself (magenta curve).

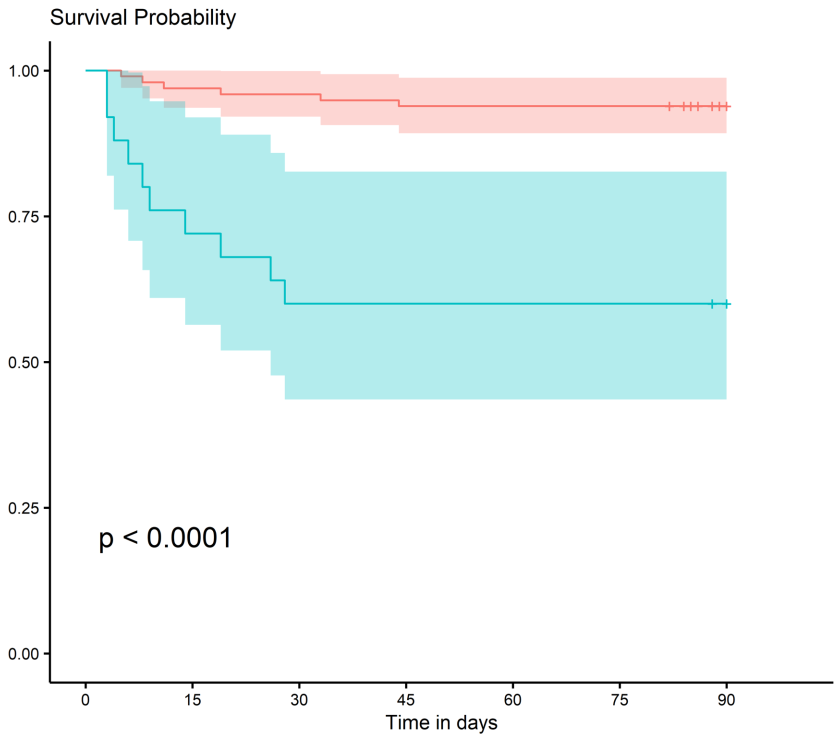


No. at risk

| | 0 | 15 | 30 | 45 | 60 | 75 | 90 |
|---|---|---|---|---|---|---|---|
| Low risk | 98 | 95 | 94 | 92 | 92 | 92 | 76 |
| High risk | 25 | 18 | 15 | 15 | 15 | 15 | 14 |

**Supplemental Figure 10**: Kaplan-Meier survival curves with 95% CIs for high- and low-risk groups in the group of patients with eGFR $\geq$ 60 mL/min/1.73 m2 at inclusion.

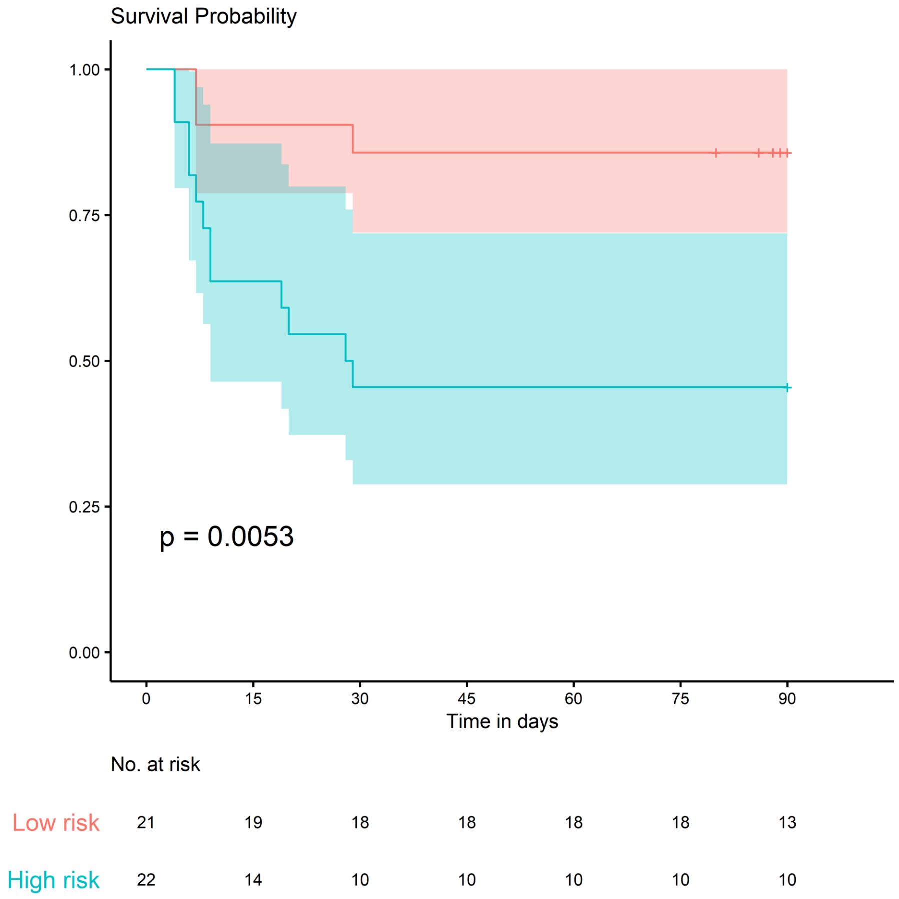


**Supplemental Figure 11**: Kaplan-Meier survival curves with 95% CIs for high- and low-risk groups in the group of patients with eGFR < 60 mL/min/1.73 m2 at inclusion.

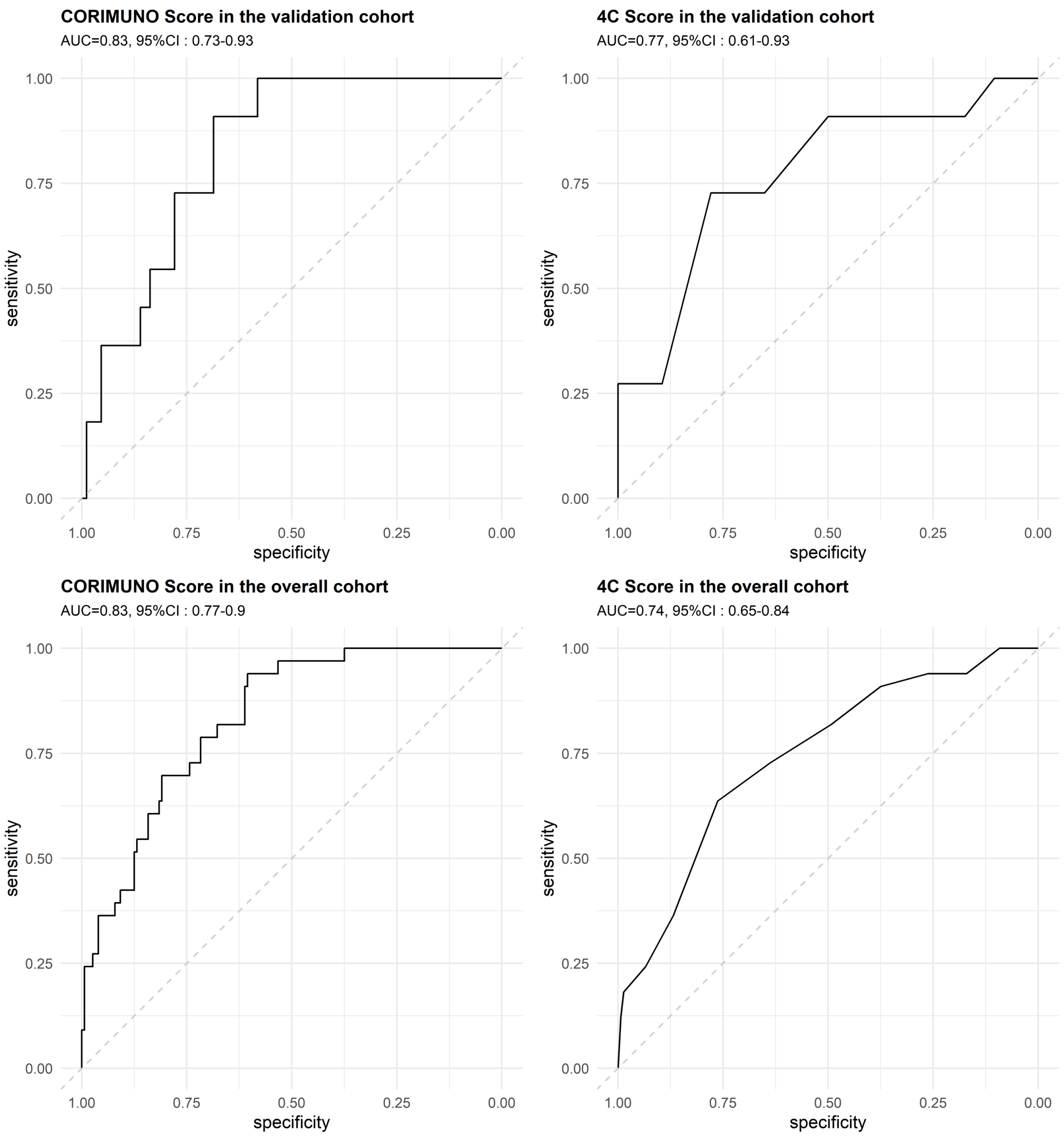


**Supplemental Figure 12**: respective ROC curves and AUC for the CORIMUNO-SCORE and the ISARI-4C score in the independent validation cohort (upper panels) and in the overall cohort (lower panels).

**Supplemental Table 1**: Serum concentration of immune mediators, cytokines, and markers of kidney and endothelial injury in the CORIMUNO cohort. Data are expressed as median and interquartile range.

| Parameters | Values | N | Statistics |
|---|---|---|---|
| | | 196 | |
| CRP | | 194 | 155 [99.2;232.2] |
| D-dimers | | 128 | 1185 [777.5;2007] |
| Lymphocytes | | 188 | 0.9 [0.6;1.2] |
| Neutrophils | | 190 | 6 [4.5;7.9] |
| Neutrophils/Lymphocytess | | 187 | 6.9 [4.5;10.2] |
| CREAT | | 167 | 74 [56.5;102.5] |
| eGFR | | 169 | 87 [57;104.9] |
| KIM-1 | | 196 | 166 [99.8;281.5] |
| Cystatine-c | | 176 | 4116800 [1746175;11857500] |
| RBP4 | | 193 | 64025000 [22839000;179070000] |
| Lipocalin-2 | | 193 | 93864.1 [73069.2;121517.1] |
| Osteopontin | | 190 | 82092.3 [43875.2;190789.9] |
| TFF3 | | 193 | 5117.9 [2539.8;11585.2] |
| IL-6 | | 196 | 89 [36.5;170] |
| IL-6R | | 196 | 36894.5 [29284.8;47013.5] |
| sGp130 | | 196 | 320.6 [263.4;364.9] |
| Granzyme B | | 196 | 35.5 [21;54] |
| IFNg | | 196 | 6.1 [2.3;18.7] |
| MCP-1 | | 196 | 973.5 [561.2;1516.5] |
| TNF$\alpha$ | | 196 | 22.6 [17.4;29.4] |
| CXCL10-IP10 | | 193 | 580.7 [237.3;1313.4] |
| IL-1a | | 196 | 0 [0;0] |
| IL-1b | | 196 | 0.4 [0;0.8] |
| IL-1RA | | 195 | 2548 [1301.5;4705.5] |
| IL-8 | | 196 | 50.4 [30.5;80.4] |
| IL-10 | | 196 | 16.2 [10.3;30.5] |
| IL-17A | $< 2.1$ | 179 | 91.33 % |
| | $\geq 2.1$ | 17 | 8.67 % |
| suPAR | | 192 | 4263.8 [1603.6;6241.1] |
| Granzyme A | | 192 | 64.9 [36.5;91.7] |
| Endoglin | | 192 | 1927.1 [1347.3;2873.2] |

| Parameters | Values | N | Statistics |
|---|---|---|---|
| PLGF | | 192 | 34.2 [21.9;74.7] |
| bFGF | | 192 | 21.6 [11.6;37.9] |
| ICAM-1 | | 192 | 526674.6 [379449.7;730821.4] |
| VCAM-1 | | 192 | 4118150 [2277950;5783775] |
| L-Selectin | | 190 | 550725.5 [411730.2;739683.8] |
| E-Selectin | | 192 | 30883.6 [22805.5;40457.2] |
| P-Selectin | | 192 | 52680.5 [36341.2;84014.2] |
| VEGF | | 192 | 198.3 [111.3;360.6] |
| sVEGFR1 | | 192 | 270.8 [204.3;379] |
| vWF | | 191 | 55697.1 [25904.3;92225.8] |
| PDGF-AA | | 191 | 4635 [2867.7;7647.5] |
| PDGF-BB | | 191 | 7928.9 [4179.4;15180.8] |
| Clusterin | | 177 | 640740000 [362910000;1021900000] |

**Supplemental Table 2**: Univariate comparison of values measured on day 1 in groups of patients with or without worsening of the WHO clinical progression score by day 14. Data are expressed as median and interquartile range.

| Parameters | N | Statistics | N | Statistics | p-value |
|---|---|---|---|---|---|
| | 140 | **No WHO score worsening** | 47 | **WHO score worsening** | |
| CRP | 138 | 142 [91.8;221.8] | 47 | 174 [128.5;245] | 0.048 |
| D-dimers | 89 | 1190 [780;1880] | 33 | 1450 [730;2770] | 0.35 |
| Lymphocytes | 134 | 0.9 [0.6;1.2] | 45 | 0.8 [0.6;1] | 0.014 |
| Neutrophils | 136 | 5.7 [4.4;7.8] | 45 | 6.6 [5.3;9.9] | 0.062 |
| Neutrophils/Lymphocytes | 133 | 6.2 [4.4;9.4] | 45 | 8.3 [5.9;17.2] | 0.0007 |
| CREAT | 117 | 70 [54;94] | 43 | 93 [68.5;141] | 0.001 |
| eGFR | 119 | 92 [68.5;105.5] | 43 | 74 [41;92.5] | 0.004 |
| KIM-1 | 140 | 145.5 [96.6;244.2] | 47 | 226 [120.5;336.5] | 0.022 |
| Cystatine-c | 124 | 3609000 [1711000;10300000] | 43 | 10260000 [2662000;16050000] | 0.021 |
| RBP4 | 138 | 64350000 [23870000;173200000] | 46 | 62570000 [22710000;184500000] | 0.60 |
| Lipocalin-2 | 138 | 92880 [70700;118900] | 46 | 96470 [80010;145500] | 0.15 |
| Osteopontin | 137 | 79260 [43800;162900] | 44 | 119200 [55060;226700] | 0.10 |
| TFF3 | 138 | 4688 [2392;10770] | 46 | 6098 [3374;12740] | 0.040 |
| IL-6 | 140 | 73.9 [25.8;142.5] | 47 | 142 [86.9;272.5] | <0.0001 |
| IL-6R | 140 | 37550 [29450;47700] | 47 | 36880 [24630;44510] | 0.23 |
| sGp130 | 140 | 324.2 [268.2;369.1] | 47 | 304.3 [256.3;347.6] | 0.063 |
| Granzyme B | 140 | 34.1 [20.9;50.4] | 47 | 37.3 [22.1;57.8] | 0.62 |
| IFNg | 140 | 6.1 [2.3;19.3] | 47 | 6.7 [2.3;17.6] | 0.94 |
| MCP-1 | 140 | 854.5 [531.8;1370] | 47 | 1238 [932;1807] | 0.001 |
| TNF$\alpha$ | 140 | 22.7 [17.4;29] | 47 | 23.3 [17.4;32.2] | 0.71 |
| CXCL10-IP10 | 138 | 507.6 [172.6;1022] | 46 | 991.4 [484.1;2273] | 0.0006 |
| IL-1a | 140 | 0 [0;0] | 47 | 0 [0;0] | 0.32 |
| IL-1b | 140 | 0.4 [0;0.7] | 47 | 0.5 [0;0.9] | 0.058 |

| Parameters | | N | Statistics | N | Statistics | p-value |
|---|---|---|---|---|---|---|
| IL-1RA | | 139 | 2225 [1204;4044] | 47 | 3995 [2300;6904] | <0.0001 |
| IL-8 | | 140 | 47 [26.6;73.6] | 47 | 58.3 [38.5;107] | 0.020 |
| IL-10 | | 140 | 13.2 [9.4;21.6] | 47 | 29.8 [18.4;41.8] | <0.0001 |
| IL-17A | < 2.1 | 126 | 90 % | 45 | 95.74 % | 0.37 |
| | ≥ 2.1 | 14 | 10 % | 2 | 4.26 % | |
| suPAR | | 138 | 4144 [1603;5997] | 45 | 5321 [2313;7159] | 0.077 |
| Granzyme A | | 138 | 67.5 [39.4;93.2] | 45 | 57.1 [8.4;81.2] | 0.033 |
| Endoglin | | 138 | 1986 [1350;2846] | 45 | 2246 [1341;3093] | 0.46 |
| PLGF | | 138 | 34.3 [21.6;72.9] | 45 | 34.8 [25.6;81] | 0.35 |
| bFGF | | 138 | 22 [12.2;39.5] | 45 | 24.8 [9.8;39.9] | 0.97 |
| ICAM-1 | | 138 | 525000 [409600;706500] | 45 | 587900 [359100;858100] | 0.61 |
| VCAM-1 | | 138 | 3704000 [2084000;5435000] | 45 | 5432000 [4034000;7567000] | 0.001 |
| L-Selectin | | 138 | 573200 [416800;802400] | 43 | 521500 [364200;657700] | 0.055 |
| E-Selectin | | 138 | 30780 [22620;41180] | 45 | 34340 [24050;44030] | 0.33 |
| P-Selectin | | 138 | 53180 [37430;87260] | 45 | 57480 [36260;75390] | 0.43 |
| VEGF | | 138 | 220.2 [123.4;381.8] | 45 | 170.9 [91.1;300.1] | 0.11 |
| sVEGFR1 | | 138 | 267.2 [204.2;351.5] | 45 | 310.9 [210.1;438.2] | 0.17 |
| vWF | | 138 | 52220 [26360;86120] | 44 | 68540 [40380;137300] | 0.055 |
| PDGF-AA | | 138 | 5233 [3231;8764] | 44 | 3803 [2313;5041] | 0.015 |
| PDGF-BB | | 138 | 9257 [4872;17710] | 44 | 7218 [2892;10660] | 0.026 |
| Clusterin | | 125 | 532800000 [356900000;959600000] | 43 | 924200000 [472300000;1.129e+09] | 0.023 |

**Supplemental Table 3**: Univariate comparison of values measured on day 1 in groups of patients who subsequently survived or died within the next 90 days. Data are expressed as median and interquartile range.

| Parameters | | N | Statistics | N | Statistics | p-value |
|---|---|---|---|---|---|---|
| | | 161 | **Death at D90: No** | 35 | **Death at D90: Yes** | |
| CRP | | 159 | 145 [93;220.5] | 35 | 183 [135;255.5] | 0.037 |
| D-dimers | | 105 | 1044 [770;1830] | 23 | 1830 [1086;2880] | 0.025 |
| Lymphocytes | | 154 | 0.9 [0.6;1.2] | 34 | 0.7 [0.6;0.9] | 0.013 |
| Neutrophils | | 156 | 5.8 [4.3;7.8] | 34 | 6.7 [5.5;9.2] | 0.096 |
| Neutrophils/Lymphocytes | | 153 | 6.4 [4.4;9.4] | 34 | 8.8 [5.6;16.6] | 0.003 |
| CREAT | | 136 | 71 [55;94] | 31 | 100 [75;175] | 0.0009 |
| eGFR | | 138 | 91.4 [69.2;105.8] | 31 | 62 [31.5;86] | 0.0004 |
| KIM-1 | | 161 | 140 [91.8;251] | 35 | 269 [170;356] | 0.0001 |
| Cystatine-c | | 143 | 3756000 [1744000;10650000] | 33 | 6765000 [1943000;20180000] | 0.17 |
| RBP4 | | 158 | 67730000 [23550000;187300000] | 35 | 55380000 [21390000;161600000] | 0.80 |
| Lipocalin-2 | | 158 | 91140 [70700;114400] | 35 | 113400 [84690;161400] | 0.002 |
| Osteopontin | | 157 | 79260 [38690;166800] | 33 | 139100 [53160;264200] | 0.076 |
| TFF3 | | 158 | 4522 [2369;9503] | 35 | 10220 [4452;13750] | 0.002 |
| IL-6 | | 161 | 78.3 [28.7;147] | 35 | 142 [82;393.5] | 0.0002 |
| IL-6R | | 161 | 36900 [29600;47780] | 35 | 36880 [24630;43220] | 0.19 |
| sGp130 | | 161 | 324.1 [269.3;367.5] | 35 | 302.9 [231.7;350.3] | 0.051 |
| Granzyme B | | 161 | 35.6 [21.5;54.5] | 35 | 35.2 [20.9;47.4] | 0.58 |
| IFNg | | 161 | 6.1 [2.4;18.5] | 35 | 5.1 [2.2;20.6] | 0.80 |
| MCP-1 | | 161 | 896 [536;1415] | 35 | 1217 [908.5;2336] | 0.005 |
| TNF$\alpha$ | | 161 | 22.2 [17.3;28.1] | 35 | 26.3 [19.2;36.9] | 0.10 |
| CXCL10-IP10 | | 158 | 521.2 [192.3;1120] | 35 | 1005 [456;2550] | 0.005 |
| IL-1a | | 161 | 0 [0;0] | 35 | 0 [0;0] | 0.71 |
| IL-1b | | 161 | 0.4 [0;0.7] | 35 | 0.5 [0;0.9] | 0.22 |
| IL-1RA | | 160 | 2410 [1212;4270] | 35 | 3995 [2354;6904] | 0.0006 |
| IL-8 | | 161 | 45.9 [28.4;76.9] | 35 | 58.6 [43.1;99.7] | 0.031 |
| IL-10 | | 161 | 14.2 [9.6;24.5] | 35 | 29.8 [18.2;41.8] | <0.0001 |
| IL-17A | < 2.1 | 147 | 91.3 % | 32 | 91.43 % | 1 |

| Parameters | | N | Statistics | N | Statistics | p-value |
|---|---|---|---|---|---|---|
| | ≥ 2.1 | 14 | 8.7 % | 3 | 8.57 % | |
| suPAR | | 157 | 4038 [1545;5941] | 35 | 5436 [2883;7198] | 0.039 |
| Granzyme A | | 157 | 67.5 [39.5;99.1] | 35 | 37.5 [11.3;78.3] | 0.002 |
| Endoglin | | 157 | 1910 [1350;2873] | 35 | 2138 [1332;2793] | 0.88 |
| PLGF | | 157 | 33.8 [21.5;73.2] | 35 | 38.4 [25.6;77.8] | 0.27 |
| bFGF | | 157 | 21.6 [11.7;37.5] | 35 | 20.3 [11;37.5] | 0.87 |
| ICAM-1 | | 157 | 516200 [374600;704500] | 35 | 664000 [452700;960700] | 0.077 |
| VCAM-1 | | 157 | 3922000 [2200000;5640000] | 35 | 4925000 [3685000;7492000] | 0.027 |
| L-Selectin | | 156 | 573200 [423800;769200] | 34 | 456000 [307900;613600] | 0.005 |
| E-Selectin | | 157 | 30310 [22020;39900] | 35 | 34340 [25210;46950] | 0.17 |
| P-Selectin | | 157 | 52450 [36260;85550] | 35 | 53520 [38260;79920] | 0.83 |
| VEGF | | 157 | 219.1 [122.8;381.2] | 35 | 123 [75.1;247.6] | 0.005 |
| sVEGFR1 | | 157 | 265.6 [203.7;347.9] | 35 | 311.8 [224.4;449.7] | 0.060 |
| vWF | | 157 | 53840 [25740;90150] | 34 | 68500 [26090;119600] | 0.22 |
| PDGF-AA | | 157 | 5045 [3068;8437] | 34 | 3803 [2255;5256] | 0.049 |
| PDGF-BB | | 157 | 8123 [4515;17010] | 34 | 7457 [3341;10550] | 0.11 |
| Clusterin | | 144 | 595800000 [363800000;994500000] | 33 | 737600000 [360200000;1.095e+09] | 0.52 |

**Details of the CORIMUNO-19 Collaborative Group**

**Writing Committee**

Olivier Hermine*, Xavier Mariette*, Pierre-Louis Tharaux*, Tabassome Madjlessi Simon, Matthieu Resche-Rigon, Raphael Porcher§, Philippe Ravaud§,

*, § equal contribution

**Steering Committee**

Olivier Hermine, Xavier Mariette, Philippe Ravaud, Pierre-Louis Tharaux, Maxime Dougados, Raphael Porcher, Matthieu Resche-Rigon, Serge Bureau, Annick Tibi, Tabassone Simon

**Scientific Committee**

Olivier Hermine (chair), Valérie Pourcher-Martinez, Karine Lacombe, Joseph Emmerich, Matthieu Mahevas, Jacques Cadranel, Frédéric Schlemmer, Muriel Fartoukh, Frédéric Pene, Bertrand Guidet, Elie Azoulay, Pierre-Louis Tharaux, Philippe Ravaud, Xavier Mariette, Yazdan Yazdanpanah , Marc Humbert, Maxime Dougados, Matthieu Resche-Rigon, Annick Tibi, Serge Bureau

**Methodology and statistics**

***Responsible for methodology***: Philippe Ravaud, Raphael Porcher, Gabriel Baron, Elodie Perrodeau

**Data Monitoring Committee**

Deepak L Bhatt (Chair), Cristina Mussini, Patrick Yeni, Galea Sandros, Kevin Winthrop, Frank Harrel.

**CORIMUNO-19 Central Coordinating Office: DRCI – AP-HP**

***Responsible for the sponsor***: Serge Bureau

Damien Vanhoye, Cécile Kedzia, Coralie Villeret, Joséphine Braun, Didier Bouton, Riad Baameur, Emmanuelle Liegey, Amel Ouslimani, Isabelle Vivaldo, Lauren Demerville, Zouleikha Bentoumi, Anne Gysembergh-Houal, Alexandre Bourgoin, Sarra Dalibey, Hélène Brocvielle, Florent Viguier , Katya Touat, Céline Le Galludec, Stephany Pong, Jeremy Combet, Aymen Rabai, Sana Bouriche, Yousra Labidi, Sofiane Mokrani, Valentin Hosansky, René Bun, Ferhat Chikhi, Sofya Mortaki, Laure Plat , Nesine Mechouar, Toufik Taib, Priscilla Andriamiandrisoa, Anne Noah, Julie Tequi-Lebras, Christine Figuerola, Sabrina Leveau

**CORIMUNO-19 platform trials Coordinating Office: Coordinator of the Clinical Research Platform of East of Paris UPMC-Paris 06 University**

***Clinical Trial Unit Lead***: Tabasome Simon

***Trial logistics***: Elodie Drouet, Gladys Aratus, Mohamed El Amine Belhout, Katia Ledra, Lucie Lampert, Samira Aklil, Marion Bolzoni, Ghizlène Tabet Derraz, Mélanie Yon, Bassem Abdelmoumen

**Drug supply: AGEPS – AP-HP**

***Pharmacy lead:*** Annick Tibi

Robin Charreteur, Céline Dupré, Kévin Cardet, Blandine Lehmann, Kamyl Baghli

**REACTING Group (Coordination between AP-HP, Inserm and Universities)**

***Chair***: Yazdan Yazdanpanah

Claire Madeleine, Eric D'Ortenzio, Oriane Puechal, Caroline Semaille

**Local clinical centers CORIMUNO-19 trials staff (listed in order of the number of patients randomized *per* site in the totality of CORIMUNO-19 trials)**

**Local clinical centres of CORIMUNO-19 trials staff** (listed in order of the number of patients randomized per site in the totality of CORIMUNO-19 trials): Beaujon, HEGP, Bicêtre, Tenon, Saint Antoine, Aulnay sous Bois, Bichat, Clermont Ferrand, Hôpital Américain de Paris, Henri Mondor, Compiègne, IGR, Cochin, Avicenne, Cayenne, Nantes, Necker, Strasbourg, Marseille, La croix saint Simon, CHOG, Foch, Versailles, Antony, Saint Denis, Lariboisière.

**AP-HP, Hôpital Beaujon, Université de Paris**

***PI:*** Yann Nguyen
***Investigators:*** Victoire de Lastours, Mathieu Uzzan, Virginie Zarrouk, Geoffrey Rossi, Annabelle Pourbaix, Annick Hamon, Olivier Roux, Victor Damas, Agnès Lefort, Vasco Honsel, Emmanuel Weiss, Anaïs Codorniu
***Local Clinical Research unit:*** Roza Rahli, Naura Gamany, Agathe Claveirole, Alexandre Navid, Tiffanie Fouque, Yonathan Cohen, Maya Lupo, Constance Gilles,
***Pharmacy:*** Zeina Louis

**AP-HP, Hôpital Européen Georges Pompidou, Université de Paris,**
***PI:*** Jean-Benoit Arlet, ***Co-Pi:*** Jean-Luc Diehl
***Investigators :*** Florence Bellenfant, Geoffroy Volle, Adrien Michon, Anne Blanchard, Alexandre Buffet, Bernard Cholley, Antoine Fayol, Edouard Flamarion, Anne Godier, Thomas Gorget, Sophie-Rym Hamada, Caroline Hauw-Berlemont, Jean-Sébastien Hulot, David Lebeaux, Marine Livrozet, Adrien Michon, Arthur Neuschwander, Marie-Aude Pennet, Benjamin Planquette, Brigitte Ranque, Olivier Sanchez, Geoffroy Volle
***Local Clinical Research unit :*** Walid Namaoui, Djamal Khimoud, Sandrine Briois, Mathias Cornic, Virginie Elisee, Jesuthasan Denis, Juliette Djadi-Prat, Pauline Jouany, Ramon Junquera, Mickael Henriques, Amina Kebir, Isabelle Lehir, Jeanne Meunier, Florence Patin, Valérie Paquet, Anne Tréhan, Véronique Vigna
***Pharmacy :*** Brigitte Sabatier
***Biological resource centre, Clinical Investigation Centre,* PARCC, Inserm:** Damien Bergerot, Charlène Jouve, Camille Knosp, Olivia Lenoir, Nassim Mahtal, Léa Resmini.

**AP-HP, Hôpital Bicêtre, Université Paris-Saclay**
***PI:*** Xavier Mariette, ***Co-Pi:*** Laurent Savale
***Investigators*** : Léliat Escaut, Mathilde Noaillon, Stephan Pavy,Céline Labeyrie, Alexandre Darmoy, Mary Soudani, Samuel Bittoun, Alicia castro Gordon, Anatole Harrois, Samy Figueiredo, Jacques Duranteau, Nadia Anguel, Arthur Pavot, Xavier Monnet, Christian Richard, Jean-Louis Teboul, Philippe Durand, Pierre Tissieres, Mitja Jevnikar, Marc Humbert, David Montani, Stephan Pavy, Nicolas Noel, Olivier Lambotte, Stephane Jauréguiberry, Elodie Baudry, Christiane Verny, Edouard Lefevre, Mohamad Zaidan
***Local Clinical Research unit***: Domitille Molinari, Gaël Leprun, Alain Fourreau, Laurent Cylly, Lamiae Grimaldi, Ikram Ramdhani, Rachida Issoufali, Camille Hostachy, Cécile Greaud
***Local Clinical Research team***: Myriam Virlouvet, Ramdane Meftali, Solène Fabre, Marion Licois, Asmaa Mamoune, Yacine Boudali
***Pharmacy:*** Clotilde Le Tiec
***Biological resource centre***: Céline Verstuyft, Anne-Marie Roques

**AP-HP, Hôpital Tenon, Université Paris-Sorbonne**
***PI:*** Sophie Georgin-Lavialle, ***Co-Pi:*** Jacques Cadranel and Gilles Pialoux

***Investigators*** : Patricia Senet, Angèle Soria, Antoine Parrot, Hélène François, Nathalie Rozensztajn, Emmanuelle Blin, Pascaline Choinier, Juliette Camuset, Jean-Simon Rech, Antony Canellas, Camille Rolland-Debord, Nadège Lemarié, Nicolas Belaube, Marine Nadal, Martin Siguier, Camille Petit-Hoang, Julie Chas, Christina Palacios, Ludovic Lassel, Juliette Bravais, Ruxandra Calin, Marwa Bachir, Guillaume Voiriot, Aude Gibelin, Michel Djibré, Vincent Labbé et Clarisse Blayau.
***Local Clinical Research unit*** : Elodie Drouet, Matthieu Lemoine, Audrey Phibel, Lucie Aunay, Eliane Bertrand, Sylviane Ravato, Marie Vayssettes, Anne Adda-Lievin, Mouniya Mebarki, Samira El-Wiyadi, Celine Wilpotte, Pélagie Thibaut, Sonia Ould Younes.
***Pharmacy*** : Julie Fillon, Isabelle Debrix
***Biological resource centre***: Soraya Fellahi, Jean-Philippe Bastard, Guillaume Lefèvre

**AP-HP, Hôpital Saint-Antoine, Université Paris-Sorbonne**
***PI:*** Arsene Mekinian**,** ***Co-PI:*** Karine Lacombe, Bertrand Guidet
***Investigators:*** Noémie Abisror, Amir Adedjouma, Diane Bollens, Marion Bonneton, Nathalie Bourcicaux, Anne Bourrier, Maria Chauchard Thibault Chiarabiani, Dorothée Chopin, Jonathan Cohen, Ines Devred, Bruno Donadille, Olivier Fain, Geoffrey Hariri, Vincent Jachiet, Patrick Ingliz, Louis schaeffer, Marc Garnier, Marc Gatfosse, Etienne Ghrenassia, Delphine Gobert, Bertrand Guidet, Jessica Krause le Garrec, Cecilia Landman, Jean Remy Lavillegrand, Benedicte Lefebvre, Thibault Mahevas, Sandie Mazerand, Jean Luc Meynard, Marjolaine Morgand, Zineb Ouazène, Jerome Pacanowski, Sébastien Riviere, Philippe Seksik, Harry Sokol, Heithem Soliman, Nadia Valin, Thomas Urbina
***Local Clinical Research unit:*** Chloé McAvoy, Maria Pereira Miranda, Zahia Benaissa, Maria Pereira, Gladys Aratus, Laurence Berard, Tabassome Simon
***Pharmacy :*** Anne Daguenel Nguyen, Elise Girault, Clémentine Mayala-Kanda, Marie Antignac, Céline Leplay
***Biological resource centre:*** Gladys Aratus, Laurence Berard, Tabassome Simon

**Centre Hospitalier Robert Ballanger - Aulnay-sous-Bois**
***PI*:** Helene Gros
***Investigators:*** Hélène Guillot, Benjamin Rossi, Marie-Anne Bouldouyre
***Local Clinical Research unit:*** Céline Nemeth

**AP-HP, Hôpital Bichat, Université de Paris**
***PI:*** Xavier Lescure, ***Co-PI:*** Jade Ghosn
***Investigators***: Odile Fleurot, Lio Collias, Bao-Chau Phung, Antoine Bachelard, Anne Rachline, Valentina Isernia, Bao-chau, Phung, Dorothée Vallois, Aurelie Sautereau, Catherine Neukrich, Antoine Dossier, Raphaël Borie, Bruno Crestani, Gregory Ducrocq Philippe Gabriel Steg, Philippe Dieude, Thomas Papo
***Local Clinical Research unit:*** Lynda Oualit, Estelle Marcault, Marhaba Chaudhry, Charlène Da Silveira, Annabelle Metois, Ismahan Mahenni, Meriam Meziani, Cyndie Nilusmas

***Local Clinical Research team:*** Sylvie Le Gac, Awa Ndiaye, Françoise Louni, Malikhone Chansombat, Zelie Julia, Solaya Chalal, Lynda Chalal
***Pharmacy:*** Laura Kramer, Jeniffer Le Grand
***Biological resource centre***: Kafif Ouifiya, Valentine Piquard, Sarah Tubiana

**CHU de Clermont-Ferrand - Gabriel Montpied**
***PI*:** Elisabeth Coupez and Kevin Grapin
***Investigators***: Claire Dupuis, Léo Sauvat, Magalie Vidal, Clément Théis, Natacha Mrozek, Olivier Lesens
***Local Clinical Research unit***: Mireille Adda, Frédéric Duée, Delphine Martineau
***Pharmacy***: Lise Bernard

**Hôpital Américain de Paris**
***PI :*** Christophe Rapp
***Investigators***: Nicholas Joza, Stephane Lasry
***Local Clinical Research unit***: Paul Ihout

**AP-HP, Hôpital Henri Mondor, Université Paris-Est Créteil**
***PI:*** Marc Michel
***Investigators***: Henri Guillet, Nicolas Limal, Adrien Gally, Sébastien Gallien, Etienne Crickx, Benjamin Le Vavasseur, Emmanuelle Kempf, Karim Jaffal, William Vindrios, Julie Oniszczuk, Matthieu Mahevas, Constance Guillaud, Fréderic Schlemmer, Pascal Lim, Elena Foïs, Giovanna Melica, Marie Matignon, Maud Jalabert, Jean-Daniel Lelièvre
***Local Clinical Research unit:*** Lina Itrani, Aurélie Baudin, David Schmitz, Marion Bourhis, Sylia Belazouz, Laetitia Languille, Caroline Boucle, Nelly Cita, Agnés Didier, Fahem Froura, Katia Ledudal, Thiziri Sadaoui
***Pharmacy***: Alaki Thiemele, Delphine Le Febvre De Bailly, Muriel Carvhalo Verlinde

**Centre Hospitalier de Compiègne**
***PI :*** Anne-Sophie Lecapitaine
***Investigators***: Mathilde Tonnelier
***Local Clinical Research unit***: Jean-Christophe Seghezzi, Sabine Brunet

**Institut Gustave Roussy, Université Paris-Saclay**
***PI:*** Jean-Marie Michot
***Investigators***: Annabelle Stoclin, Emeline Colomba, Fanny Pommeret, Christophe Willekens
***Local Clinical Research unit***: Rosa Da Silva, Valérie Dejean, Yasmina Mekid, Ines Ben-Mabrouk, Ferdaous sahli
***Pharmacy***: Florence Netzer
***Biological resource centre***: Caroline Pradon, Laurence Drouard, Valérie Camara-Clayette

**AP-HP, Hôpital Cochin, Université de Paris**
***PI:*** Nathalie Costedoat-Chalumeau **Co-PI**: Liem Binh Luong, Frédéric Pene
***Investigators***: Solidad Henriquez, Zakaria Ait Hamou, Célia Azoulay, Sarah Benghanem, Philippe Blanche, Nicolas Carlier, Benjamin Chaigne, Remy Gauzit, Hassan Joumaa, Mathieu Jozwiak, Marie Lachâtre, Hélène Lafoeste, Odie Launay, Paul Legendre, Jonathan Marey, Caroline Morbieu, Lola-Jade Palmieri, Tali-Anne Szwebel
***Local Clinical Research unit:*** Hendy Abdoul, Alexandra Bruneau, Audrey Beclin-Clabaux, Charly Larrieu, Pierre Montanari, Eric Dufour
***Local Clinical Research team***: Ada Clarke, Catherine Le Bourlout, Nathalie Marin, Nathalie Menage, Samira Saleh-Mghir, Mamadou Salif Cisse, Kahina Cheref
***Pharmacy***: Corinne Guerin, Jérémie Zerbit

**AP-HP, Hôpital Avicenne, Université Paris-Nord Sorbonne**
***PI:*** Luca Semerano
***Investigators*** : Sébastien Abad, Ruben Bénainous, Nicolas Bonnet, Celine Comparon, Yves Cohen, Hugues Cordel, Robin Dhote, Nathalie Dournon, Boris Duchemann, Nathan Ebstein, Thomas Gille, Benedicte Giroux-Leprieur, Jeanne Goupil de Bouille, Hilario Nunes, Johanna Oziel, Dominique Roulot, Lucile Sese, Claire Tantet, Yurdagul Uzunhan.
***Local Clinical Research Unit:*** Coralie Bloch-Queyrat, Vincent Levy, Fadhila Messani, Mohammed Rahaoui, Mylène Petit, Rawan El-Hajj,Miassa Bentifraouine.
***Pharmacy***: Sabrina Brahmi, Vanessa Rathoin, Marthe Rigal

**CHU Cayenne**
***PI:*** Félix Djossou
***Investigators*** : Claire Rouzaud, Anne Rachline, Laurene Deconinck, Lucas Perez, Mathilde Boutrou, Rachline Anne
***Local Clinical Research Unit:*** Audrey Phibel, Mayka Mergeay-Fabre
***Pharmacy***:

**CHU de Nantes**
***PI:*** François Raffi
***Investigators*** : Maeva Lefebvre, Colin Deschanvres, Raphaël Lecompte
***Local Clinical Research Unit:*** Alexandre Duval, Jérémie Orain, Morgane Le Bras, Anne-Sophie Lecompte
***Pharmacy***:

**AP-HP, Hôpital Necker Enfants Malades, Université de Paris**
***PI:*** Claire Aguilar
***Investigators*** : Emmanuel Laffont, Alexandra Serris, Fanny Alby-Laurent, Carole Burger, Clara Campos-Vega, Nathalie Chavarot, Benjamin Fournier, Claire Rouzaud, Damien Vimpère

***Local Clinical Research unit***: Caroline Elie, Prissile Bakouboula, Laure Choupeaux, Sophie Granville, Elodie Issorat
***Pharmacy***: Christine Broissand
***Biological resource centre***: Marie-Alexandra Alyanakian

**Hôpitaux Universitaires de Strasbourg, Université de Strasbourg**
***Co-PIs:*** Jacques-Eric Gottenberg and Yves Hansmann
***Investigators:*** François Danion, Yvon Ruch, Gabriela Gautier Vargas, Frédéric Blanc, Sophie Ohlmann-Caillard, Vincent Castelain, Emmanuel Chatelus, Eva Chatron, Olivier Collange, François Danion, Frédéric De Blay, Pierre Diemunsch, Sophie Diemunsch, Renaud Felten, Bernard Goichot, Valentin Greigert, Aurelien Guffroy, Bob Heger, Charlotte Kaeuffer, Loic Kassegne, Anne Sophie Korganow, Pierrick Le Borgne, Nicolas Lefebvre, Paul-Michel Mertes, Eric Noll, Mathieu Oberlin, Vincent Poindron, Julien Pottecher, Yvon Ruch, François Weill
***Local Clinical Research unit:*** Nicolas Meyer, Emmanuel Andres, Eric Demonsant, Hakim Tayebi, Gabriel Nisand, Stéphane Brin, Cédric Sublon
***Pharmacy :*** Guillaume Becker, Anne Hutt, Tristan Martin
***Biological Resource Centre****:* Sophie Bayer, Catherine Metzger

**Hôpital Marseille - Hôpital Saint Joseph**
***PI***: Boris Bienvenu
***Investigators:*** Victor Lancon, Antoine Poulet, Christina Audoly
***Local Clinical Research unit***: Laurence Lecomte, Kristina Beziriganyan, Belkacem Asselate, Rania Benfrej
***Pharmacy***: Laure Allanic, Elena Kiouris, Marie-Hélène Legros, Christine Lemagner, Pascal Martel, Vincent Provitolo

**Groupe Hospitalier Diaconesse Croix Saint-Simon**
***PI:*** Jonathan London
***Investigators:*** Wladimir Mauhin, Clara Mellot, Rolland Amathieu, Gabriel Le jour
***Local Clinical Research unit:*** Younes Keroumi, Celine Chapel
***Pharmacy:*** Emmanuelle Guillot

**Centre Hospitalier de l'ouest Guyanais**
***PI:*** Franklin Samou Fantchou
***Investigators:***
***Local Clinical Research unit:*** Audrey Phibel
***Pharmacy:*** Emmanuelle Guillot

**Hôpital Foch- Suresnes**
***PI***: Félix Ackermann
***Investigators:*** Hélène salvator, Mathilde Roumier
***Local Clinical Research unit***: Kewin Panel, Mathilde Le Marchand
***Pharmacy***: Aurélie Chan Hew Wai, Dimitri Fremont

**CH André Mignot-Versailles**
***PI***: Antoine Gros
***Investigators***: Roderich Meckenstock
***Local Clinical Research Unit***: Estelle Henry, Anaïs Beulaygue, Salima Kaoudji
***Pharmacy***: Claire Courtin, Anne Pattyn

**Hôpital Privé d'Antony**
***PI***: Alexandre Morel,
***Investigators***: Gilles Garcia, Abolfazl Mohebbi
***Local Clinical Research unit***: Férial Berbour, Mélanie Dehais,
***Pharmacy:*** Anne-Lise Pouliquen, Alison Klasen, Loren Soyez-Herkert

**Hôpital Delafontaine de Saint Denis**
***PI:*** Nicolas Gambier
***Local Clinical Research unit***: Stéphanie Cossec

***Pharmacy:*** Thierno Dieye
***Local Clinical Research unit***: Stéphanie Cossec

**AP-HP, Hôpital Lariboisière, Université de Paris**
***PI:*** Damien Sène
***Investigators*** : Ruxandra Burlacu, Benjamin Chousterman, Bruno Mégarbanne, Pascal Richette, Jean-Pierre Riveline, Aline Frazier
***Local Clinical Research unit*** : Eric Vicaut, Laure Berton, Tassadit Hadjam, Miguel Alejandro Vazquez-Ibarra, Clément Jourdaine, Olivia Tran, Véronique Jouis
***Pharmacy*** : Aude Jacob, Julie Smati, Stéphane Renaud
***Biological resource centre***: Claire Pernin, Lydia Suarez

**Acknowledgements**
We thank for their support people having participated to the study by different aspects

**CORIMUNO-19 Central Coordinating Office: DRCI – AP-HP**
*Officers from DRCI:* Coralie Villeret, Joséphine Braun, Didier Bouton, Riad Baameur, Emmanuelle Liegey, Amel Ouslimani, Isabelle Vivaldo, Zouleikha Bentoumi, Hélène Brocvielle, Florent Viguier , Katya Touat, Céline Le Galludec, Stephany Pong, Jeremy Combet, Aymen Rabai, Sana Bouriche, Yousra Labidi, Sofiane Mokrani, Valentin Hosansky, René Bun, Ferhat Chikhi, Sofya Mortaki, Laure Plat , Nesine Mechouar, Toufik Taib, Priscilla Andriamiandrisoa, Anne Noah, Julie Tequi-Lebras, Christine Figuerola, Sabrina Leveau

**Local clinical centers CORIMUNO-19 trials staff**

**AP-HP, Hôpital Bicêtre University Hospital and Université Paris-Saclay**
***Clinicians having taken care of patients*****:** Jean-Louis Teboul, Laurent Guerin, Astrid Bertier, Soufia Ayed, Arthur Pavot, Thibault Creutin, Tai Pham, Frédéric Desmoulins, Matthieu Guillet, Manon Dekeyser, Olivier Sitbon, Etienne Marie Jutant, David Montani, Xavier Jais, Jérémie Pichon, Athenais Boucly, Sophie Bulifon, Andrei Seferian, Antoine Beurnier, Florence Parent, Roseline D'Oiron, Antoine Cheret, Jeremy Gottlieb, Jérémie Benichou, Alexandre Dormoy, Céline Labeyrie, Julien Henry, Alexandre Virone
***Clinical Research technicians, clinical research doctors* and students:** Myriam Virlouvet & Solène Fabre (Gynaecology-obstetrics dpt), Yacine Boudali, Sylvie Miconnet (Rheumatology dpt), Marie-Thérèse Legaud (Nephrology dpt), Jugurtha Berkenou (Plateforme Maladies Rares), Anne Brel, Andreea Catina, Céline Chevreau, Jodie Ferrand, Marie Galopeau, Noémie Monard, Clémence Roche, Cypria Siva, Béatrice Vest, Kévin Hakkakian, Peter Chen, Lucile Durand, Jeremie Robinsohn, Yohann Cauche, Simon Rigaux, Léa Delarue, Claire Carles, Céline Koeberle, Baptiste Desjardins, Thomas Boesch, Nicolas Leroux, Pauline Potisek, Christian Luna, Khalis Sarah, Chabbert Jerôme, Bourdin Joachim, Lola Othily, Constans Fanny, Rosalie Brabant, Nassim Benaissa, Alexandre Khuu, Ariane Guillaume, Neslon Droguete Gomes, Djilali Batouche, Clémence Mille, Camille Hostachy Arnaud Peramo, Marc Labetoulle, Antoine Rousseau

**AP-HP, Hôpital Tenon and Université Paris-Sorbonne**
***Clinicians having taken care of patients*****:** Vincent Fallet, Ludovic Lassel, Gilles Grateau, Gilles Pialoux, Eric Bouvard, François Lionnet, Sarah Mattioni, Claude Bachmeyer, Aline Santin, Muriel Fartoukh, Guillaume Voiriot, Michel Djibré, Sophie Le Nagat, Julie Chas, Martin Siguier, Léa Savey, Olivier Steichen, Elzbieta

Garandeau, Hélène Goulet, Lise Matton, Pierre Rigaud, Enora Berti, Clarisse Blayau, Alexandre Elabbadi, Aude Gibelin, Julien Lopinto, Paris Meng, Matthieu Turpin.
***Clinical Research technicians, clinical research doctors, students:*** Cora Lucet, Jean-Edouard Martin, Kaïna Mahmoudi, Sara E Sa, Aude Dos Santos, Marine Moreau, Tamazouzt Ribert, Céline Wilpotte, Marie Prades-Borio, Nathalie Sermondade.

**Hôpitaux de Strasbourg and Université de Strasbourg**
***Clinicians having taken care of patients*: ** Lydia Calabrese, Maxence Meyer, Carmen Suna
***Clinical Research technicians*** Lamia Amoura, Khaled Benneddif, Marjory Berns, Anne Botzung, Thouma-La Chanthavinout, Stéphane Hecketsweiler, Joël Herrmann, Fanny Huselstein, Luz Marc, Corine Martin, Jessica Parthonneau, Lucie Rauch, Nathalie Reinbold, Arnaud Romoli, Danielle Roy, Elisabeth Vergne, Yazhuo Gong, Sara Zgheib

**AP-HP, Hôpital Saint-Antoine and Université Paris Sorbonne**
***Clinicians having taken care of patients:*** Ingrid Reinhart, Olivia Pietri et Marie Lequoy, Philippe Nuss, Eric Maury, Hafid Ait Oufella, Abelkrim Abdelmalek, Laurent Beaugerie, Armelle Poujol, Nicolas Carbonnel, Lionel Arrivé, Clément Cholet, Laurence Monnier Cholley, Franck Boccara, Ariel Cohen, Sarou Adavane, Alice Courties, Sonia Alamowitch, Edouard Januel, Jean Capron, Marion Yger, Fabienne Fieux, Jean Luc Baudel, Dominique Pateron, Jennifer Sobotka, Pierre Alexis Raynal, Olivier Cha, Eda Bui, Murielle Chaillet, Christelle Hermand, Helene Vallet, Valery Bellamy, Laura Moisi, Caroline Thomas, Emmanuel Pardo, Lucie Darrivère, Laure Bottin
***Clinical Research technicians and students:*** Cyrielle Letaillandier, Manuela LeCam, Christian Tran, Jean-Luc Lagneau, Julie Lamarque

**AP-HP, Hôpital Européen Georges Pompidou and Université de Paris**
***Clinicians having taken care of patients*: ** Sébastien Clerc, Elsa Denoix, Thomas Gorget, Jean Pastre, Marie-Aude Penet, Jérôme Pinot, Claire Potencier, Matthieu Le Melledo, Amer Hamdan
***Biology platform:*** Eric Tartour, Marie-Agnès Dragon-Durey, Franck Pages, Véronique Frémeaux-Bacchi, Stéphanie Baron
***Clinical Research technicians and students:*** Assya AKLI, Shahnaze AQIL, Leslie Benattar, Sérine Chaibi ; Louise Chantelot ; Valentin Demeure, Anna Dordonnat, Marin Durand, Alexandre Ifrah, Camille Lavril, Anouk Giulianelli, Alexandre Mary, Salomé Oliviera, Xavier Pinus, Pierre Poujard, Marguerite Requillard, Anouchka Ron, Matéo Sanchis-Borja ; Adèle Sandot ; Gabrielle Stevenin, Heloise Wauquiez

**AP-HP, Hôpital Bichat and Université de Paris**
Véronique Joly, Sylvie Lariven, Christophe Rioux, Diane Le Pluart, Laurenne Deconinck, Sophie Ismael, Marie Gilbert, Anne Gervais Hassan Tharini, Agathe

Bounhiol, Mayda Al Rahi , Bérénice Souhail, Maya Husain, François Maillet thomas Volpe Simon Gressens, Aanabelle Pourbaix ,Marion Parisey, Marie Dubert, Timothée Bironne Cloé De Broucker
Simon Valayer, Jeanne Chauffier, Catherine Boussard, Axelle Fuentes, Paul Crespin, Jonathan Vermes

**AP-HP, Hôpital Beaujon and Université de Paris**
***Clinicians having taken care of patients*****:** Paer-Selim Abback, Mathieu Battelier, Marion Bedbeder, Hélène Bout, Marine Cazaux, Pierre-Marie Choinier, Félix Corre, Sonja Curac, Clémentine De La Porte Des Vaux, Louis De Mestier Du Bourg, Paul de Saint, Marco Dioguardi Burgio, Fatou Drame, Bruno Fantin, Adrien Galy, Jules Gregory, Marion Guillouet, Antoine Hamon, Gueorgui Iakovlev, Linda Khoy-Ear, Sophie Lacaille, Amandine Landrieux, Lucie Laurent, Minh-Pierre Le, Elise Mallart, Jean-Denis Moyer, Yousra Kherabi, Emma Oliosi, Simon Raynal, Vinciane Rebours, Isabelle Rennuit, Trystan Sebastianutti, Damien Soudan, Carmen Stefanescu, Xavier Treton, Tristan Thibault Sogorb, Thomas Vauchel, Eve Garrigues, Audrey le Bot, Hadrien Kimseng, Hafsah Hachad, Hélène Gouze, Sabryne Berkani, Lucile Doucet, Amre Shalaby, Gabriel Kemoun, Vinca Montmeat, Elise Rambaud, Anne-Sophie Gordon
***Clinical Research technicians* and students:** Kahina Lamrani, Massissilia Krouchi, Souhila Laceb, Lynda Lagha, Lilit Kelesyan, Adnan Mamodaly, Laurie Leguay, Paul Pechmajou, Laure Marchal, Lydia Meziane, Fatiha Mavouna, Cécile Pavis, Julien Matricon, Nathalie Gastellier,
***Biological resource center*****:** Frédéric Bert, Katell Peoc'h, Claude Hercend, Emmanuelle De Raucourt, Catherine Trichet

**AP-HP, Hôpital Lariboisière and Université de Paris**
***Clinicians having taken care of patients*****:** Albertini Mathieu, Amador Borrero Blanca, Bouajila Sara, Britany Kimbimbi, Burlacu Ruxandra, Cacoub Léa, Champion Karine, Chauvin Anthony, Delcey Véronique, Depond Audrey, Dillinger Jean-Guillaume, Feron Florine, Frazier Aline, Thomas Funck-Bretano, Galland Joris, Gauthier Diane-Cecile, Gautier Jean-François, Henry Patrick, Huscenot Tessa, Sarah Izabel Mathilde, Jaulerry, Jouabli Moenes, Julla Jean-Baptiste, Kevorkian Jean-Philippe, Laloi Michelin Marie, Leroy Pierre, Lopes Amanda, Mangin Olivier, Michon Maxime, Mouly Stephane, Munier Anne-Lise, Nahmani Yoram, Nicol Martin, Nicolas Eroan, Poulat Audrey, Revue Eric, Richette Pascal, Riveline Jean-Pierre, Rubenstein Emma, Sellier Pierre-Olivier, Sene Damien, Thoreau Benjamin, Vodovar Dominique, Zanin Adrien, Aveneau Clément, Bastard Paul, Beauvais Diane, Boghez Loredana, Borderiou Alix, Conway Paul, Cosma Lavignia, Davy Vincent, Desjardin Clément, Devatine Sandra, Ducroz Gerardin Christel, Dupe Charlotte, Gobert Chloé, Gros Clotilde, Kadiri Soumaya, Khan Enmat, Ongnessek Sandrine, Rhmari Fatima, Sacco Isabelle, Saptefrat Natalia, Schaupp Pauline, Serre Justine, Sideris Georgios, Smati Sonia, Tournier Marine, treca Pauline, Truong Tony, Tuffier Mathilde, Arcelli

Mattéo, Boue Yvonnick, Copie Alban, Deye Nicolas, Ekherian Jean-Michel, Errabih, Zaccaria, Gonde Antoine, Grant Caroline, Guerin Emmanuelle, Magalhaes Adèle, Malissin Isabelle, Megarbane Bruno, Meurisse Edouard, Mrad Aymen, Naim Giulia, Nguyen Philippe, Nitenberg Kiyoko, Pepin-Lehalleur Adrien, Perault Arthur, Perrin Lucile, Renaud Maxime, Sutterlin Laetitia, Voicu Sebastian

**AP-HP, Hôpital Avicenne and Université Paris-Nord Sorbonne University**
***Clinicians having taken care of patients***: Olivier Bouchaud, Johann Cailhol, Simon Chauveau, Morgane Didier Marcelot, Florence Cymbalista, Soline de Monteynard, Agniezka Kolakowska, Florence Duperron, , Farid Foudi, Olivia Freynet, Florence Jeny, Warda Khamis, Sylvain Le Jeune, Marilucy Lopez Sublet, Fréderic Mechai, Elise Ouedraogo, Maxime Patout, Jaehyo Suhl, Yacine Tandjaoui-Lambiotte.
***Clinical Research technicians***: Amani Rebai, Miassa Slimani, Rawan Belmokhtar, Miassa Bentifraouine, Lina Innes Skandri, Houda Allalou, God Chancely M Bayi Matondo

**AP-HP, Hôpital Cochin and Université Descartes**
***Clinicians having taken care of patients:*** Luc Mouthon, Alexis Regent, Benjamin Terrier, Célia Azoulay
***Clinical Research technicians and students:*** Meriem Benfodda, Kamil Chitour, Gaelle Clavere, Jeanne Colombe, Firas Faraht, Caroline Gaudefroy, Moez Jallouli, Nathalie Menage, Alexandre Moores, Isabelle Peigney, Julie Rotureau, Mathilde Vallet, Alizée Verdon, Coralie Samba, Daniela Nylund, Casimir Jeantaud, Luka Lachiver, Sarah Larbi, Valentine Gloaguen, Emilie Chau, Patsy Vanhaesebrouck, Wendy Sok.

**AP-HP, Hôpital Henri Mondor and Université Paris-Est Créteil**
**Clinicians having taken care of patients:** Armand Mekontso Dessap, Nicolas De Prost, François Bagate, Keyvan Razazi, Philippe Le Corvoisier, Raphaele Arrouasse, Jean-Daniel Lelievre, Jean-François Deux,
***Clinical Research technicians and students:*** Mouhamed Dieng, Asunejad, Geoffrey Rossi, Charles Binette, Isabelle Veillard, Aurelie Wiedemann.

**AP-HP, Hôpital Necker Enfants Malades and Université de Paris**
***Clinicians having taken care of patients***: Nada Aboumerouane, Genevieve Afantchao, Lucile Amrouche, Dany Anglicheau, Sylvain Auvity, Melanie Brunel, Lucienne Chatenoud, Anna DiMarzio, Marine Driessen, Lionel Lamhaut , Vanessa Lopez, Perrine Parize, Laurent Sabbah, Rebecca Sberro-Soussan, Anne Scemla, Scarlett Wise, Julien Zuber
***Clinical Research technicians*** **and students:** Sylvain Goupil, Marie-Noelle Halley, Meriem Imarazene, Jouda Marouene, Fabio Mecozzi, Lilia Toumi

**Institut Gustave Roussy and Université Paris-Saclay**

Giulia Baciarello, Thomas Hueso, Alain Gaffinel, Franck Griscelli, Fabrice Barlesi, Jean-Charles Soria, Benjamin Besse, Laurence Albiges, Julie Laurence, Camille Sallee, Bertrand Gachot, Julien Hadoux, Nolwenn Lucas, Geraldine Martinez, Marie-Pollen Moulle, Isabelle Rousseau, Kahina Chetouane, Frédéric Troalen.

**Hôpital Privé d'Antony**
***Clinicians having taken care of patients*:** Gilles Garcia, Anne Herkert, Véronique Zarka, Elias Dabboura, Abolfazl Mohebbi, Michel Benhamou, Jean-Pierre Deyme, Olivier Andremont, Benoit Vandenbunder, Franck Le Queau, Jean-Charles Gagnard, Joël Livartowski, Catherine Heyraud-Blanchet.

**Hôpital de Saint-Denis**
***Clinicians having taken care of patients:*** Antoine Casel, Elisa Pasqualoni, Rita Dujon, Fanny Jouan, Stéphanie Ngo, François Lhote, Marion Dollat, Marie Poupard, Marie-Aude Khuong, Remi Lefrançois, Carole Henry, Naomi Sayre.

# Cohort Multiple randomized controlled trials, open-label of immune modulatory drugs and other treatments in COVID-19 patients

# Statistical analysis plan

## Specific version of the General Statistical Analysis Plan for MONICOR

## Biomonitoring and complex analyses in support of the open-label randomized controlled trial cohort of immunomodulatory products and other treatments for patients with COVID-19.

## 1. Administrative information

### A. General

| | |
|---|---|
| ACRONYM : | CORIMUNO-19 |
| REGISTRATION NUMBER | Sponsor code: APHP200375<br>EudraCT:<br>ClinicalTrials.gov: NCT04324047 |
| SAP VERSION & DATE | 1.0, 09/05/2020 |
| PROTOCOL VERSION: | Version 6.0 of 04/05/2020 |

### B. Roles and responsibilities

| | |
|---|---|
| TRIAL CHIEF INVESTIGATOR | Dr THARAUX Pierre-Louis |
| METHODOLOGIST | Pr RESCHE-RIGON Matthieu |
| TRIAL STATISTICIAN | WALTER-PETRICH Anouk |
| SAP AUTHOR | |
| DATA-MANAGER | |
| OTHER CONTRIBUTORS | |

### C. SAP revision history

| Version | Date | Author | Description of changes |
|---|---|---|---|
| 1.0 | 09/05/2020 | | Creation of SAP |
| | | | |
| | | | |
| | | | |
| | | | |
| | | | |
| | | | |
| | | | |

## 2. Introduction

### A. Rationale of the study/Hypothesis of the research

A significant challenge to meta-analyzing the various and numerous clinical trials in the field of COVID-19 is to confidently delineate the population enrolled in clinical trials, as they differ markedly in terms of mortality, despite efforts to standardize the assessment of clinical progression scores by the World Health Organisation and empiric comparisons of routine inflammatory markers and comorbidities. Thus, given the uncertainty about stratifying patients with COVID-19, considerable interest exists in risk stratification scores to support frontline clinical decision-making. Available risk stratification tools have a high risk of bias, a small sample size resulting in uncertainty, poor reporting, and a lack of formal validation.

To develop a tool to address these challenges, we will conduct multiparameter molecular analyses in a well-defined patient cohort and replicate them in an independent cohort. Such molecular analyses encompassed markers of acute kidney injury (AKI), systemic inflammation, and endothelial alterations.

We will evaluate whether an early measurement of a panel of inflammation, endothelial, and AKI serum markers is associated with the risk of critical COVID-19 pneumonia and death.

**The exploratory set of markers aims at evaluating three groups of biological variables that may reflect the mechanisms or consequences of organ injury and/or represent therapeutic targets**:

1- **Inflammatory mediators**: the interleukin-6 system (serum concentration of IL-6, soluble Interleukin 6 RA (sIL6RA), soluble gp130), the interleukin-1 system (interleukin 1 alpha (IL1a), interleukin 1 beta (IL1B)), interleukin 17A (IL17A), interferon-gamma (IFNg), C-X-C motif chemokine ligand 10 (CXCL10)/Interferon gamma-induced protein 10 (IP-10), granzyme A and granzyme B, IL-8, IL-10, CCL2/ monocyte chemoattractant protein-1 (MCP-1), interleukin 1 RA (IL1RA), tumor necrosis factor-alpha (TNF$\alpha$), myeloperoxydase (MPO).

2- **Markers of endothelial injury or of an inflammatory vascular phenotype**:

Proteins involved in endothelial maintenance: Vascular Endothelial Growth Factor A (VEGFA), soluble VEGF Receptor 1 (sVEGFR1), Platelet-derived Growth Factor AA (PDGF-AA), PDGF-BB, soluble endoglin (sEng), Placental Growth Factor (PlGF), Basic Fibroblast Growth Factor (bFGF),

and of vascular injury: soluble E-selectin, soluble P-selectin, soluble L-selectin, soluble urokinase plasminogen activator (sUPAR), soluble InterCellular Adhesion Molecule 1 (sICAM-1), soluble Vascular Cell Adhesion Molecule 1 (sVCAM-1), soluble von Willebrand Factor (vWF), endothelin-1 (ET-1), Clusterin.

3- We will combine the above-cited analytes with **markers of kidney dysfunction (estimated glomerular filtration rate (eGFR) and injury**: serum concentrations of Cystatin C, Retinol Binding protein 4 (RBP4), Lipocalin 2 (LCN2)/neutrophil

gelatinase-associated lipocalin (NGAL), Osteopontin, and Trefoil Factor 3 (TFF3) and kidney injury molecule-1 (KIM-1)/HAVCR.

We hypothesize that severe and critical forms of COVID-19 pneumonia may be associated with uncontrolled activation of the immune system, microvascular injury and AKI, the latter condition being a known risk factor for high morbidity and mortality in a wide array of conditions, including sepsis.

### B. Objectives

#### I. Primary Objective

To identify and validate a composite score based on biomarkers measured at baseline that predicts 90-day mortality, allowing classification of patients into distinct risk categories (e.g., high-risk vs. low-risk of death).

#### II. Secondary Objectives

To identify biomarkers associated with an increase of more than one point in the WHO Clinical Progression Scale [CPS] within 14 days after randomization.

#### III. Exploratory Objectives

- To identify specific markers associated with 90-day mortality.
- To assess the predictive value and discriminative ability of the composite score for survival within each treatment arm (Tocilizumab, Sarilumab, or Usual care)
- To identify biological clusters of patients at Day 1

## 3. Study methods

### A. Study design & framework

This is an exploratory, retrospective analysis combining individual participant data from two randomized controlled trials evaluating Tocilizumab and Sarilumab in patients with COVID-19 (CORIMUNO-TOCI and CORIMUNO-SARI) and biomarker measurements.

### B. Randomization

Randomization procedures were conducted within each parent trial according to their original protocols. In this analysis, treatment allocation and trial identifiers will be retained for subgroup analyses. No new randomization or reassignment will occur, and the original allocation structure will be respected for all treatment-related analyses.

### C. Sample size justification

Given the exploratory nature of the MONICOR study, it is not possible to infer a minimum number of subjects required. Nonetheless, 1/ several biomarkers that we will test have shown significant association with critical forms of CORIMUNO-19 (sUPAR, IL-6, vWF, sICAM-1) in smaller studies. 2/ The CORIMUNO-TOCI trial suggests that tocilizumab administration to

the studied population may have reduced the risk of non-invasive ventilation, mechanical ventilation, or death by day 14. This may reflect a differential biological profile.

3/ We will use samples collected in the CORIMUNO-SARI trial (NCT04324073) as a training cohort. We will next use an independent external dataset (CORIMUNO-TOCI trial (NCT04331808) to improve the generalisability of our study.

### D. Statistical interim analyses and stopping guidance

No interim analyses or stopping rules are planned. Both parent trials have completed data collection and primary analyses. This exploratory work will be performed on finalized, locked datasets, with no sequential testing or alpha adjustment.

### E. Timing of final analysis

All outcomes will be analyzed collectively after data harmonization and integration of all biomarker datasets. Assessments will be conducted at predefined time points: Day 1 (baseline), Day 14, and Day 90. All analyses will use the finalized dataset; any subsequent reanalyses will be considered post-hoc and documented separately.

## 4. Statistical principles

### A. Confidence intervals and P-values

Associations with binary outcomes will be quantified using Odds Ratios (ORs), with Wald confidence intervals computed on the natural logarithm of the OR. The area under the ROC curve (AUC) will be estimated, and a confidence interval will be calculated using the DeLong method. Time-to-event outcomes will be evaluated by comparing survival curves, and statistical significance will be assessed using the log-rank test.

A bilateral type I error rate of 5% without multiplicity adjustment will be applied to the analyses.

### B. Analysis populations

All analyses will be performed in complete cases: all eligible participants with available biomarker and outcome data.

## 5. Trial population

### A. Eligibility

**Inclusion Criteria for the CORIMUNO-19 cohort:**

1. Laboratory-confirmed SARS-CoV-2 infection as determined by PCR, or other commercial or public health assay in any specimen and/or CT Scan before randomization (Following typical radiological findings (ground glass abnormalities,

and absence of lymphadenopathy, pleural effusion, pulmonary nodules, lung cavitation)

2. Hospitalized patients
3. Illness of any duration and severity (mild, moderate, severe, critical, see annexe 1), with symptoms (fever, cough, respiratory difficulties, shortness of breath), and at least one of the following:
    a. Radiographic infiltrates by imaging (CT scan)
    b. Clinical assessment (evidence of rales/crackles on exam or respiratory rate >25/min) AND SpO2≤94% on room air
    c. SpO2≤97 % with O2 > 5L/min or Respiratory rate>=30/min
    d. Requiring mechanical ventilation
    e. With any comorbidities (TBD such as acute kidney injury, cardiovascular condition, pulmonary disease, obesity, high blood pressure, diabetes, chronic kidney diseases, haematological diseases, sickle cell diseases, autoimmune and auto-inflammatory, pregnant women, HIV infected, etc.)
4. Male or female adult ≥ 18 years of age at time of enrolment
5. Patients must be able and willing to comply with study visits and procedures.
6. Patient agrees to the collection of oropharyngeal and nasal swabs and venous blood per protocol
7. Written informed consent provided by the patient or, alternatively, by the next-of-kin before any protocol-specific procedures.

**Exclusion Criteria for the CORIMUNO-19 cohort:**

Participation in another clinical trial is not an exclusion criterion, depending on the medication. ***Patients included in the antiviral REACTING trial are not excluded, as well as patients from COVIDICUS trial.***

Severe cardiovascular disease, including acute myocardial infarction, unstable angina pectoris, coronary revascularisation procedure, congestive heart failure of NYHA Class III or IV, stroke, including a transient ischemic attack, oedema of cardiac origin and left ventricular ejection fraction ≤ 50% are not excluded and should be discussed in each therapeutic arm.

- Patients with any condition that the physician judges could be detrimental to the patient's participation in this study, including any clinically important deviations from normal clinical laboratory values or concurrent medical conditions (active infections, such as severe bacterial infections, aspergillosis, or tuberculosis, depending on the tested medication).
- Absence of Health Insurance
- Subject protected by law under guardianship or curatorship

**Inclusion Criteria for the CORIMUNO-TOCI trial:**

1. Patients included in the CORIMUNO-19 cohort

2. Patients belonging to one of the 2 following groups:

*- Group 1: patients **not requiring ICU** at admission with moderate and severe pneumopathy according to the WHO **Criteria of severity of COVID pneumopathy.***

***Moderate cases***

Cases meeting all of the following criteria:

- Showing fever and respiratory symptoms with radiological findings of pneumonia.
- Requiring between 3L/min and 5L/min of oxygen to maintain SpO2 >97%

***Severe cases***

Cases meeting any of the following criteria:

- Respiratory distress (≧30 breaths/ min);
- Oxygen saturation ≤93% at rest in ambient air, or Oxygen saturation ≤97 % with O2 > 5L/min.
- PaO2/FiO2≦300mmHg

*- Group 2: patients **requiring ICU** based on **Criteria of severity of COVID pneumopathy.***

- Respiratory failure and requiring mechanical ventilation
- No do-not-resuscitate order (DNR order)

**Exclusion Criteria for the CORIMUNO-TOCI trial:**

- Patients with exclusion criteria to the CORIMUNO-19 cohort.
- Known hypersensitivity to Tocilizumab or to any of their excipients.
- Pregnancy
- Current documented bacterial infection
- Patient with any of the following laboratory results out of the ranges detailed below at screening should be discussed, depending on the medication:
  - Absolute neutrophil count (ANC) ≤ 1.0 x 109/L
  - Haemoglobin level: no limitation
  - Platelets (PLT) < 50 G /L

SGOT or SGPT > 5N

**Inclusion Criteria for the CORIMUNO-SARI trial:**

1. Patients included in the CORIMUNO-19 cohort

2. Patients belonging to one of the 2 following groups:

*- Group 1: patients **not requiring ICU** at admission with moderate and severe pneumopathy according to the WHO **Criteria of severity of COVID pneumopathy.***

***Moderate cases***

Cases meeting all of the following criteria:

- Showing fever and respiratory symptoms with radiological findings of pneumonia.
- Requiring between 3L/min and 5L/min of oxygen to maintain SpO2 >97%

***Severe cases***

Cases meeting any of the following criteria:

- Respiratory distress (≧30 breaths/ min);
- Oxygen saturation≤93% at rest in ambient air, or Oxygen saturation ≤97 % with O2 > 5L/min.
- PaO2/FiO2≦300mmHg

*- Group 2: patients **requiring ICU** based on **Criteria of severity of COVID pneumopathy.***

- Respiratory failure requiring mechanical ventilation
- No do-not-resuscitate order (DNR order)

**Exclusion Criteria for the CORIMUNO-SARI trial:**

1. Patients with exclusion criteria from the CORIMUNO-19 cohort.
2. Known hypersensitivity to Sarilumab or to any of their excipients.
3. Pregnancy
4. Current documented bacterial infection
5. Patient with any of the following laboratory results out of the ranges detailed below at screening should be discussed, depending on the medication:
    a. Absolute neutrophil count (ANC) ≤ 1.0 x 109/L
    b. Haemoglobin level: no limitation
    c. Platelets (PLT) < 50 G /L

SGOT or SGPT > 5N

## C. Recruitment

Participants for this analysis will be recruited from two completed randomized controlled trials evaluating Tocilizumab and Sarilumab in patients with COVID-19 (TOCI and SARI trials).

Only patients who were randomized in either trial, have a biomarker measurement at Day 1, and have sufficient follow-up to assess the primary endpoint (90-day mortality) will be included.

No new patient recruitment will occur, as this is a retrospective secondary analysis based on existing trial data.

## D. Withdrawal/follow-up

Participant withdrawal and follow-up information will be based on data recorded in the two randomized controlled trials.

No additional follow-up will be performed for this secondary analysis.

## E. Baseline patient characteristics

| Variable | Computation (if done) | Type of variable | Measurement unit |
|---|---|---|---|
| **Baseline characteristics** | | | |
| Age | (Randomization date – Birth date) / 365.25<br>For birth date, imputation of day by 01 | Continuous | Years |
| Sex | - | Binary (Male/Female) | - |
| Weight | - | Continuous | Kg |
| BMI | - | Continuous | Kg/m$^2$ |
| WHO score | - | Binary (≥ 6/<6) | |
| RT-PCR-confirmed SARS-CoV-2 infection | - | Binary (Yes/No) | |
| Temperature | - | Continuous | C |
| Respiratory rate (bpm) | - | Continuous | Bpm |
| O2 Flow (L/min) | - | Continuous | L/min |
| SpO2 (%) | - | Continuous | % |
| Time from symptoms onset to randomization (days) | (Randomization date – Symptom onset date) | Continuous | Days |
| Time from hospital admission to randomization (days) | (Randomization date – hospital admission date) | Continuous | Days |
| Chronic cardiac disease | - | Binary (Yes/No) | - |
| Diabetes | - | Binary (Yes/No) | - |
| Chronic kidney disease (stage 1 to 3) | - | Binary (Yes/No) | - |
| Asthma | - | Binary (Yes/No) | - |
| Chronic pulmonary disease (not asthma) | - | Binary (Yes/No) | - |
| Active malignant neoplasm | - | Binary (Yes/No) | - |
| Smoking | - | Binary (Yes/No) | - |
| **Measurements** | | | |
| Platelets | - | Continuous | $10^9$/L |
| CRP | - | Continuous | mg/L |
| DDimers | - | Continuous | ng/mL |
| Lymphocytes | - | Continuous | $10^9$/L |
| Neutrophils | - | Continuous | $10^9$/L |
| Neutrophils / Lymphocytes | Neutrophils / Lymphocytes | Continuous | |
| Creatinine | - | Continuous | micromol/L |
| eGFR | - | Continuous | mL/min/1,73m2 |
| KIM-1 | - | Continuous | pg/mL |
| Cystatine C | - | Continuous | pg/mL |
| RBP4 | - | Continuous | pg/mL |
| Lipocalin-2 | - | Continuous | pg/mL |
| Osteopontin/OPN | - | Continuous | pg/mL |
| TFF3 | - | Continuous | pg/mL |
| Gp130 soluble | - | Continuous | pg/mL |
| IL-6 | - | Continuous | pg/mL |
| IL-6 receptor | - | Continuous | pg/mL |
| Granzyme A | - | Continuous | pg/mL |
| Granzyme B | - | Continuous | pg/mL |
| IFNg | - | Continuous | pg/mL |
| MCP-1 | - | Continuous | pg/mL |

| | | | |
|---|---|---|---|
| TNF alpha | - | Continuous | pg/mL |
| IL-1a | - | Continuous | pg/mL |
| IL-1b | - | Continuous | pg/mL |
| IL1-RA | - | Continuous | pg/mL |
| IL-8 | - | Continuous | pg/mL |
| IL-10 | - | Continuous | pg/mL |
| IL-17A | - | Continuous | pg/mL |
| uPAR | - | Continuous | pg/mL |
| Endoglin | - | Continuous | pg/mL |
| PLGF | - | Continuous | pg/mL |
| L-Selectin | - | Continuous | pg/mL |
| E-Selectin | - | Continuous | pg/mL |
| P-Selectin | - | Continuous | pg/mL |
| bFGF | - | Continuous | pg/mL |
| ICAM-1 | - | Continuous | pg/mL |
| VEGFA | - | Continuous | pg/mL |
| PDGF-AA | - | Continuous | pg/mL |
| PDGF-BB | - | Continuous | pg/mL |
| vWF | - | Continuous | pg/mL |
| Clusterin | - | Continuous | pg/mL |
| CXCL10-IP10 | - | Continuous | pg/mL |
| ET1 | - | Continuous | pg/mL |
| MPO | - | Continuous | ng/mL |

# 6. Analysis

## A. Outcome definitions

| Objective | Endpoint | Time of measurement | Computation (if done) | Analysis method |
|---|---|---|---|---|
| **Primary outcome** | | | | |
| To identify and validate a composite score based on biomarkers measured at baseline that predicts 90-day mortality, allowing classification of patients into distinct risk categories (e.g., high-risk vs. low-risk of death). | All-cause mortality by Day 90. | Day 90 | Composite biomarker-based risk score predicting 90-day mortality. | Biomarkers will be analyzed in the SARI cohort (training set) using Wilcoxon rank-sum tests to identify candidates associated with 90-day mortality.<br>Selected biomarkers will then be combined in a LASSO logistic regression model to derive a composite risk score.<br>The optimal cut-off will be determined using the Youden index from the ROC curve to classify patients as high-risk or low-risk.<br>Survival between risk groups will be compared by Kaplan–Meier curves and log-rank tests in both the SARI (training) and TOCI (validation) cohorts. |
| **Secondary outcome** | | | | |
| To identify biomarkers associated with an increase of more than one point in the WHO Clinical Progression Scale [CPS] within 14 days after randomization. | Clinical worsening defined as an increase of >1 point on the WHO Clinical Progression Scale (CPS) compared with baseline. | Day 14 | ΔCPS = CPS_D14 – CPS_D0; dichotomized as >1 vs ≤1 | Biomarker concentrations will be compared between patients with and without clinical worsening (ΔCPS > 1 vs ≤ 1) using Wilcoxon rank-sum tests in the pooled cohort combining the SARI and TOCI trials. |
| **Exploratory outcomes** | | | | |
| To identify specific markers associated with 90-day mortality. | All-cause mortality by Day 90 in the pooled cohort | Day 90 | | Biomarker concentrations will be compared between survivors and non-survivors at Day 90 using Wilcoxon rank-sum tests in the pooled cohort combining the SARI and TOCI trials. |
| To assess the predictive value and discriminative ability of the composite score for survival within each treatment arm (Tocilizumab, Sarilumab, or Usual care) | Overall survival up to Day 90 according to biomarker-based risk groups (high-risk vs low-risk) defined by the composite score cut-off. | D1 to Day 90 | Time from randomization to death from any cause; survivors censored at last follow-up. | Patients will be classified into high- and low-risk groups according to the biomarker-based score. Survival curves will be generated using the Kaplan–Meier method, and differences between groups will be compared using log-rank tests within each treatment arm (Tocilizumab, Sarilumab, and Usual care). |
| To identify biological clusters of patients at Day 1 | Identification of biological clusters of patients at day 1 | D1 | | Unsupervised multivariate analysis; visualization by dendrogram; correlation matrices used to explore relationships among biomarkers. |

## B. Analysis methods

Quantitative data will be described using median, range and interquartile range [IQR]. Qualitative data will be presented as frequencies and percentages (based on the non-missing sample size).

Comparisons will be performed using the Wilcoxon rank-sum test for quantitative data and the Fisher's exact test for qualitative data. We will next use regularization to control model complexity. To overcome the risk of overfitting, we will consider a multivariable model using penalized logistic regression (Least Absolute Shrinkage and Selection Operator (LASSO)) to predict death at 90 days. All variables with a P-value below 0.05 in the univariate analyses will be included in the model. The penalization parameter (lambda) will be assessed by cross-validation. Final associations will be estimated using Odds Ratios and their 95% Confidence Intervals (95% CI). Receiver operating characteristic (ROC) curves will then be generated from the linear predictor of the previous model, and the areas under the curves (AUCs) will be calculated to evaluate the model's performance on the training, validation, and combined cohorts. AUC 95% CIs will be generated using the DeLong method. The risk threshold will be determined with Youden's index. Survival and their 95% Confidence Intervals will be estimated using the Kaplan-Meier estimator and compared using log-rank tests. All tests will be two-sided at the 0.05 level.

Exploratory analyses of biomarker relationships:
Hierarchical clustering of standardized biomarker concentrations will be performed using Euclidean distance and Ward's minimum variance method to explore similarities among biomarkers, and relationships between biomarkers will be assessed using Spearman correlation coefficients.

## C. Missing data

Analyses will be performed on complete cases only; missing data will not be imputed.

## D. Statistical software

Analyses will be made using R version 4.1.0.
